# Taxonomic profiling and populational patterns of bacterial bile salt hydrolase (BSH) genes based on worldwide human gut microbiome

**DOI:** 10.1186/s40168-019-0628-3

**Published:** 2019-01-23

**Authors:** Ziwei Song, Yuanyuan Cai, Xingzhen Lao, Xue Wang, Xiaoxuan Lin, Yingyun Cui, Praveen Kumar Kalavagunta, Jun Liao, Liang Jin, Jing Shang, Jing Li

**Affiliations:** 1 School of Life Science and Technology, China Pharmaceutical University, Nanjing, 210009 China; 2 State Key Laboratory of Natural Medicines, China Pharmaceutical University, Nanjing, 210009 China

**Keywords:** Bile salt hydrolase (BSH), Gut microbiota, Taxonomic identification, Bile acids, Number of paralogs

## Abstract

**Background:**

Bile salt hydrolase plays an important role in bile acid-mediated signaling pathways, which regulate lipid absorption, glucose metabolism, and energy homeostasis. Several reports suggest that changes in the composition of bile acids are found in many diseases caused by dysbacteriosis.

**Results:**

Here, we present the taxonomic identification of bile salt hydrolase (BSH) in human microbiota and elucidate the abundance and activity differences of various bacterial BSH among 11 different populations from six continents. For the first time, we revealed that bile salt hydrolase protein sequences (BSHs) are distributed in 591 intestinal bacterial strains within 117 genera in human microbiota, and 27.52% of these bacterial strains containing BSH paralogs. Significant variations are observed in BSH distribution patterns among different populations. Based on phylogenetic analysis, we reclassified these BSHs into eight phylotypes and investigated the abundance patterns of these phylotypes among different populations. From the inspection of enzyme activity among different BSH phylotypes, BSH-T3 showed the highest enzyme activity and is only found in *Lactobaclillus*. The phylotypes of BSH-T5 and BSH-T6 mainly from *Bacteroides* with high percentage of paralogs exhibit different enzyme activity and deconjugation activity. Furthermore, we found that there were significant differences between healthy individuals and patients with atherosclerosis and diabetes in some phylotypes of BSHs though the correlations were pleiotropic.

**Conclusion:**

This study revealed the taxonomic and abundance profiling of BSH in human gut microbiome and provided a phylogenetic-based system to assess BSHs activity by classifying the target sequence into specific phylotype. Furthermore, the present work disclosed the variation patterns of BSHs among different populations of geographical regions and health/disease cohorts, which is essential to understand the role of BSH in the development and progression of related diseases.

**Electronic supplementary material:**

The online version of this article (10.1186/s40168-019-0628-3) contains supplementary material, which is available to authorized users.

## Background

Bile acids (BAs) are well known for regulating cholesterol balance, and disorders of BAs enterohepatic circulation can cause gallbladder [1] or gastrointestinal diseases [2]. The metabolism of BAs is also known to be associated with diabetes [3], obesity [4], and cardiovascular diseases [5]. BAs are synthesized from cholesterol in hepatocytes, after which they are further conjugated with the amino acids glycine or taurine to form bile salts and transfer to intestine (Additional file 1: Figure S1). Notably, the amphiphilic combination of bile salts is essential to the absorption of fat in the intestine. However, excessive bile salts are toxic to intestinal bacteria [6].

Bile salt hydrolase (BSH, EC 3.5.1.24), also designated as choloylglycine hydrolase, present in the gut microbiome can catalyze the hydrolysis of conjugated bile salts into deconjugated BAs to preserve the balance of metabolism of BAs. Moreover, deconjugated BAs serve as signaling molecules to facilitate the secretion of GLP-1 [7], activate multiple receptors [8–10], and influence different metabolic processes to cause a variety of diseases [11, 12].

BSH has already been identified in several microbial genera, including *Lactobacillus* [13, 14], *Bifidobacterium* [15], *Enterococcus* [16], *Clostridium* spp. [17], and *Bacteroides* [18]. Interestingly, *L. johnsonii* PF01 was reported to have three distinct BSHs [19], and two BSH enzymes (BSH1 and BSH2) from *Lactobacillus salivarius* LMG14476 were found to have strikingly different properties with respect to their catalytic efficiency and substrate preference [20]. The above studies indicate that, in some strains, the number of BSH genes can be variable as can their properties.

Given the important role of gut microbiota in bile acid metabolism, it is essential to systematically identify which bacterial strains hold BSH genes, as well as how their abundance and activity varies in the gut. Because of the rapid development of next-generation sequencing technologies, some public databases now provide the baselines and variances of human gut metagenomic data (e.g., the Human Microbiome Project (HMP) [21] and META genomics of the Human Intestinal Tract (MetaHIT) [22]). Additionally, different populations exhibit considerable variations in gut microbiota because of different genetic backgrounds, environments, and especially large differences in dietary habits [23], which is also helpful to the investigation of the variation patterns of BSH in human gut microbiota.

The HMP reference genome database demonstrates the taxonomic distribution of bacteria containing bile salt hydrolase protein sequences (BSHs), while the public gut metagenome databases can provide the abundance of BSHs in various populations of geographical regions and health/disease cohorts. Thus, in the present study, we investigated the taxonomic, populational, and functional patterns of BSH in human gut microbiota using computational and biological approaches with the following underlying aims: (1) to identify bacteria encoding BSHs in human gut microbiota; (2) to investigate the variation in sequence, structural, and biological activity variations of BSHs from different bacteria; (3) to explore the factors that might affect the patterns of BSHs in human microbiota; and (4) to discover the associations between the relative abundance of BSHs with several diseases.

## Results

### Sequence and structure comparisons of BSHs

**Fig. 1:**
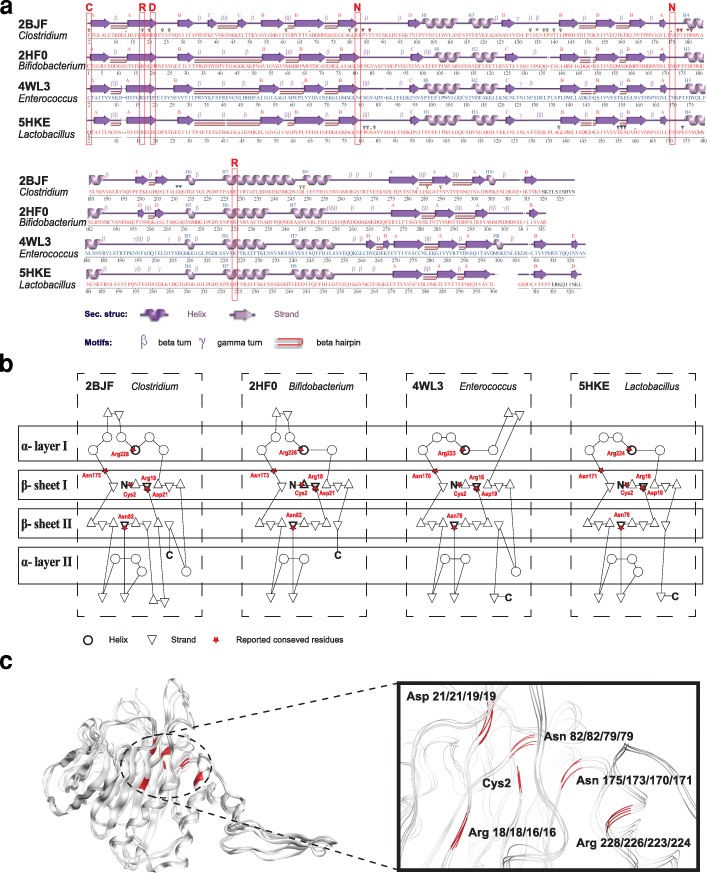
Comparison of known representative BSHs. **a** Secondary structure comparison. **b** Topological diagram. **c** 3D structure comparison of four BSHs that have reported their active site residues. Here, the red frames in the secondary structures, the red stars in the topological diagram, and the red chains in 3D structures both show the reported active site residues in four BSH

At first, we obtained all available (total 765) BSHs from the National Center for Biotechnology Information (NCBI) database [24] (Additional file 1: Figure S2a), which were found to be distributed in 69 genera, mainly *Bacillus*, *Staphylococcus*, *Paenibacillus*, *Lysinibacillus*, *Clostridium*, and *Brevibacillus*, etc. (Additional file 1: Figure S3a). However, only 12 3D structures of BSH were reported in the protein data bank (PDB) database [25] (Additional file 1: Figure S2b). Then, we compared the amino acid sequences (Additional file 1: Figure S4) and structures (Fig. 1) of four BSHs that have reported their active site residues (Additional file 2: Table S1). The four BSH were from *Clostridium* [26], *Bifidobacterium* [27], *Lactobacillus* [28], and *Enterococcus* [29], respectively.

The molecular weights of the BSH subunits ranged from 28 to 50 kDa [30]. Comparison of sequences revealed that the number of amino acids in BSHs from *Clostridium*, *Bifidobacterium*, *Lactobacillus*, and *Enterococcus* were 329, 316, 325, and 326, respectively (Additional file 1: Figure S4a). Pairwise amino acid sequence alignments of the BSHs showed that the highest identity was 54.29% (comparison of BSHs from *Lactobacillus* and *Enterococcus*), and the lowest identity was 34.91% (comparison of BSHs from *Bifidobacterium* and *Enterococcus*) (Additional file 1: Figure S4b). Overall, their average value of sequence identity was 40.41% (Additional file 1: Figure S4a).

The secondary structures (the pattern of hydrogen bonds between the amino hydrogen and carboxyl oxygen atoms in the peptide backbone) of these BSH showed that the six reported active site residues were conservative (Fig. 1a, highlight by red frame), although the number of α-helix and β-strand were different. The topological diagram of BSH showed that the domain had a six-layered structure of composition βαββαβ, and the core of BSH was composed of two sandwiched antiparallel α-sheets, which were conformed to the structure characteristics of the Ntn-hydrolase family [29, 31] (Fig. 1b).

According to previous studies, the conserved and functional important residues in these four BSH were Cys2, Arg18/18/16/16, Asp21/21/19/19, Asn82/82/79/79, Asn175/173/170/171, and Arg228/226/223/224 [26–29]. These six residues were closed in the 3D structure to form the core active site of BSH although they were far apart in BSH (Fig. 1c). It is noteworthy that the functional active sites in BSHs are not only these six residues. For instance, there were totally 30 residues that are contacted with various substrates in BSH of 2BJF (Additional file 1: Figure S5, Additional file 2: Table S1).

These results indicated that although there exist much sequence differences among different BSH, the functional residues, αββα fold of secondary structure, and core active site are conserved.

### Taxonomic identification and paralogs investigation of BSHs

**Fig. 2:**
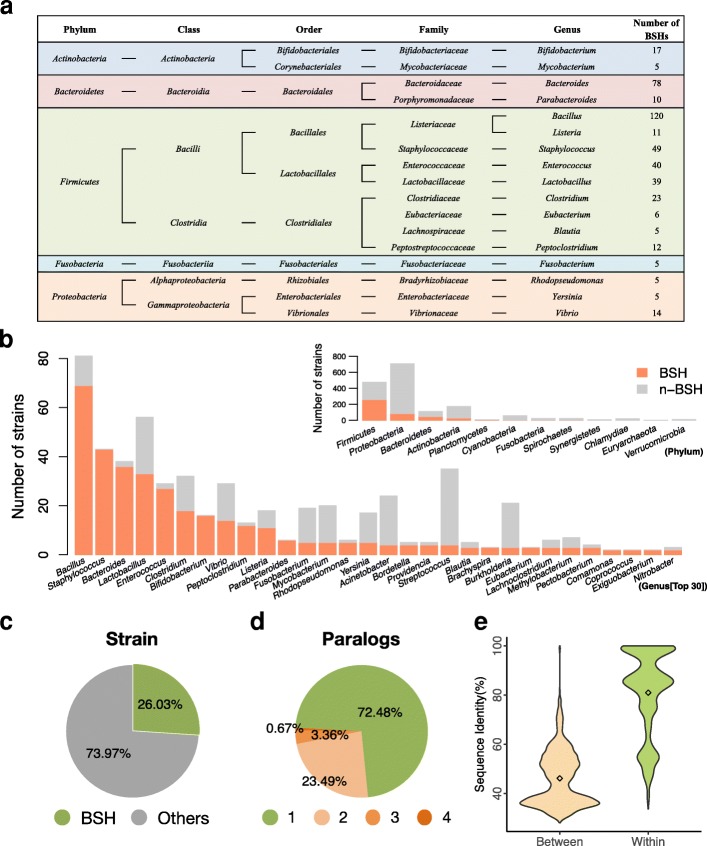
Taxonomic characterization of BSHs obtained from the HMP. **a** Taxonomic identification of BSHs in different main genera, i.e., containing more than five BSHs. **b** Quantity of BSHs containing bacteria stains at the phylum and genus level, respectively. **c** The proportion of strains expressing BSHs among all strains from the HMP database. **d** The proportion of different paralogs in all BSHs from the HMP database. **e** Density distribution of BSHs between and within genera in the HMP database. Details are shown in Additional file 2: Table S2

To explore differences among all BSHs from bacteria in human microbiota, a total number of 591 BSHs were identified from the HMP database [32] (Additional file 2: Table S2). All these BSHs were assigned to 117 genera from 12 phyla, namely *Actinobacteria*, *Bacteroidetes*, *Euryarchaeota*, *Firmicutes*, and *Proteobacteria*, etc. (Additional file 2: Table S2, Fig. 2). Among these, more than half of BSHs (353 sequences, 59.73%) containing bacteria belonged to *Firmicutes* (Additional file 2: Table S2, Fig. 2a). Almost all strains in these genera, e.g., *Staphylococcus*, *Bacteroides*, *Enterococcus*, *Bifidobacterium*, *Peptoclostridium*, and *Parabacteroides*, contained BSHs (Fig. 2b). The results showed that a total of 26.03% of bacteria strains in the HMP microbiota reference genome encoding BSH (Fig. 2c). Furthermore, these 591 BSHs were distributed in only 447 strains because of the existence of paralogs (Additional file 2: Table S2, Additional file 1: Figure S6). Evaluation of the proportions of different numbers of paralogs (NP) revealed that 72.48% (324 strains) of strains encoding only one BSH, 23.49% (105 strains) had two, 3.36% (15 strains) had three BSHs, and three strains of *Bacteroides* had four BSHs (Fig. 2d, Additional file 2: Table S2). In total, there are 20 genera of bacteria in which 123 strains have paralogs (Addtional file 1: Figure S7 and Additional file 2: Table S2). Among them, *Bacillus*, *Bacteroides*, and *Enterococcus* have highest number of strains with paralogs (46, 27, and 13, respectively; Additional file 1: Figure S7).

The homology of BSHs between genera demonstrated that they were mainly distributed in two intervals, one at about 35% and the other at about 50% (Fig. 2e). However, there was also a lower identity of BSHs within the genus at about 50% (Fig. 2e). These results indicated that distinguishing BSHs by genera might not be a rational method because of the paralogs of BSHs harbored in the bacterial genome.

### Populational comparison and multivariable-adjusted analyses of BSHs

The BSHs obtained from the HMP database were further screened in the gut microbiome of worldwide populations (Additional file 2: Table S3). Among the 561 BSHs we obtained from the HMP database, only 156 were presented in the gut microbiome of 591 people from 11 different countries of six continents (Additional file 2: Table S4). The drastic decrease in the number of strains (591 to 156, 26.40%) indicates that many BSH-expressing bacteria are very low in abundance in the human gut microbiome and undetectable under the current sequencing depth.

**Fig. 3:**
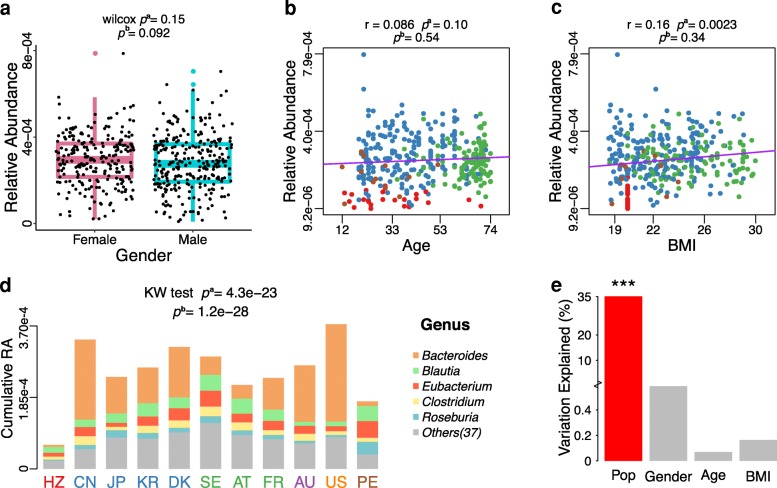
Effects of multiple factors on relative abundance of BSHs. **a** Relative abundance (RA) of BSHs in different genders among nine populations. **b** Correlation between BSHs RA and age in eight populations. **c** Correlation between BSHs RA and BMI in seven populations. **d** The cumulative RA and genera distribution of BSHs in different populations. **e** The multivariable-adjusted analysis of cumulative RA of BSHs against individual conditions including four factors. *p*^a^ values were calculated by nonparametric univariate method (Mann-Whitney *U* test) corrected for false discovery rate. *p*^b^ values were calculated by multivariable-adjusted analyses using linear regression model for target factor with remaining three factors. Here, the 11 populations form six continents include Africa: HZ; Asia: CN, JP, and KR; Europe: DK, SE, AT, and FR; Oceania: AU; North America (N-America): US; South America (S-America): PE. Note that the datasets of KR and AU have no individual information of gender, age, and BMI, and the dataset of HZ has no individual information of BMI, while the dataset of US has no individual information of age and BMI. Thus, the above datasets were excluded in related analysis. Details are shown in Additional file 2: Table S3, S4

Considering the influence of several factors among individuals, we compared differences in the distribution of BSHs based on age, gender, and body mass index (BMI) by using a single factor analysis (Fig. 3). No significant differences were found in the relative abundance (RA) of BSHs in the human gut microbiota between males and females (Fig. 3a, *p*^*b*^ = 0.092). Further, correlation analysis revealed a very weak relationship between the RA of BSH and age (Fig. 3b, *p*^*b*^ = 0.54) or BMI (Fig. 3c, *p*^*b*^ = 0. 34) of individuals.

Based on the cumulative RA distribution of BSHs in different populations (Fig. 3d, Additional file 2: Table S4), the America (US) population exhibited the highest cumulative RA of BSHs (3.74 × 10^−4^), while the Hadza ethnic group of Tanzania (HZ) exhibited the lowest (6.20 × 10^−5^). The lower cumulative RA of BSHs in the HZ population may have been because they live in habitats almost completely isolated from other humans and have had relatively little modification to their basic way of life for hundreds of years [33]. Moreover, majority of BSHs were identified from five genera, *Bacteroides*, *Blautia*, *Eubacterium*, *Clostridium*, and *Roseburia*, which represented about 71.31% of the total abundance of BSHs in human gut metagenomes (Fig. 3d, Additional file 2: Table S5).

Multivariable regression analysis was implemented to evaluate the relationship of BSHs abundance with gender, age, BMI, and population (Fig. 3). Gender, age, and BMI were not significantly correlated with the abundance of BSHs after adjustment (Fig. 3a, b, and c). However, significant associations were found between the cumulative RA of BSHs and populations from different geographical regions (*p*^*b*^ = 1.2e-28, Fig. 3d). The populational factor explained the highest variations (35.15%) of BSHs abundance among the four factors (Fig. 3e). These results were consistent with the observation that disparity of the gut microbiota composition in different populations was mainly caused by geography [34].

### Reclassification and variation patterns of BSHs

**Fig. 4:**
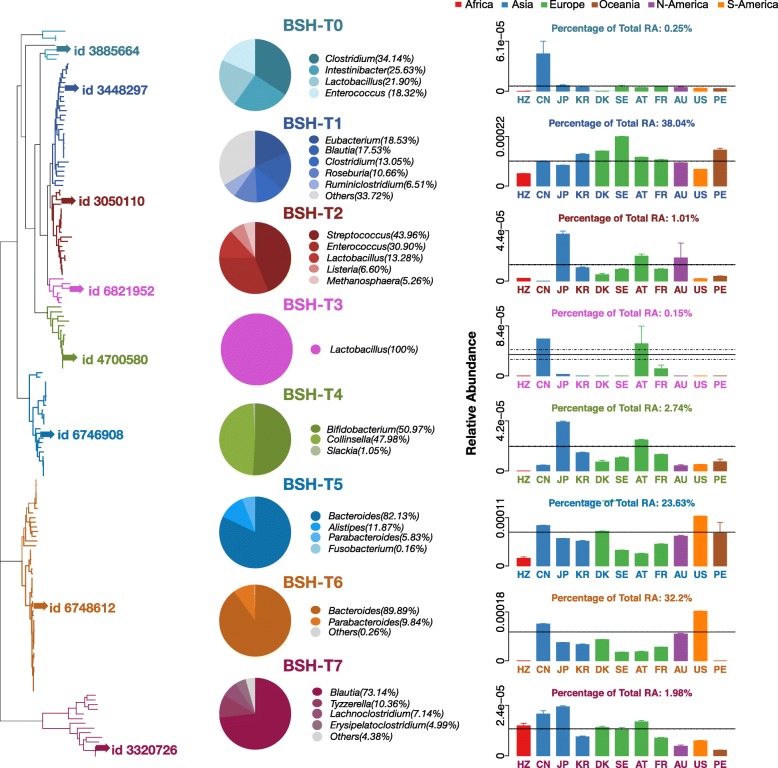
Taxonomic characterization and populational patterns of reclassified BSHs. The left panel shows the phylogenetic tree of BSHs in 11 populations from six continents, include Africa: HZ; Asia: CN, JP, and KR; Europe: DK, SE, AT, and FR; Oceania: AU; North America (N-America): US; South America (S-America): PE. Different colors represent the eight reclassified BSH phylotypes. The highlighted sequences with “id” were selected for molecular docking and enzyme activity experiments. The pies in the middle panel showed the percentage of genera in each phylotype of BSHs (for genera in group “others,” please refer to Additional file 2: Table S5). Bar plots in the right panel indicate the relative abundance (RA) of each BSH phylotypes in different populations. Details are shown in Additional file 2: Table S4–S6

Given the BSHs within genera showed a broad range of sequence dissimilarity because of the paralogs of BSHs in many strains (Fig. 2d, Additional file 1: Figure S7), the genus level patterns of BSHs abundance might not reflect the functional variations and phenotype associations clearly. Thus, we reclassified the 156 BSHs in the gut microbiome of populations observed in this study (11 countries, six continents) into eight phylotypes based on a phylogenetic tree (left panel of Fig. 4, Additional file 1: Figure S8). The results revealed seven major phylotypes, BSH-T1 (including 27 BSHs from 24 strains), BSH-T2 (including 19 BSHs from 17 strains), BSH-T3 (including six BSHs from five strains), BSH-T4 (including 14 BSHs from 14 strains), BSH-T5 (including 23 BSHs from 23 strains), BSH-T6 (including 46 BSHs from 39 strains), and BSH-T7 (including 14 BSHs from 14 strains). Moreover, BSH-T0 (including seven BSH from one to seven strains) could not be classified by hierarchical algorithms, but their sequences were relatively close to each other on the phylogenetic tree.

Among the genera distribution (middle panel of Fig 4, Additional file 2: Table S4) and population patterns (right panel of Fig. 4, Additional file 2: Table S4) of each phylotype, BSH-T0 contained only 0.25% of the RA of BSHs, which were not shown in the gut microbiome of HZ population and distributed in *Clostridium*, *Intestinibacter*, *Lactobacillus*, and *Enterococcus*, respectively. BSH-T1 contained 38.04% of the total RA of BSHs, which were mainly distributed in *Eubacterium*, *Blautia*, *Clostridium*, *Roseburia*, and *Ruminococcus* and could be found in the gut microbiota of all 11 populations. BSH-T2 contained 1.01% of the total RA of BSHs, and the major genera of this phylotype were *Streptococcus* and *Enterococcus*. Notably, this phylotype was not found in gut microbiota of the population of China (CN; right panel of Fig 4, Additional file 2: Table S6). Sequences of BSH-T3 were all from *Lactobacillus*, which were only found in CN, Japan (JP), Austria (AT), and France (FR) populations and showed higher RA in CN and AT populations (Fig. 4, Additional file 2: Table S6). BSH-T4 represented 2.74% of the total RA of BSHs, which were mainly distributed in *Bifidobacterium* and *Collinsella* and could not be found in HZ population (Fig. 4, Additional file 2: Table S6). The RA of BSH-T4 in gut microbiota of the population of JP was higher than that of other populations (Fig. 4, Additional file 2: Table S6). BSH-T5 contained 23.63% of the total RA of BSHs, which were mainly distributed in *Bacteroides* (Fig. 4). BSH-T6 contained 32.2% of the total RA of BSHs, which were also mainly distributed in *Bacteroides* (Fig. 4). However, there were no BSHs from this phylotype could be found in HZ and Peru (PE) population (Fig. 4, Additional file 2: Table S6). BSH-T7, which contained 1.98% of the total RA of BSHs mainly distributed in *Blautia* and could be found in the population from all countries (Fig. 4, Additional file 2: Table S6).

There were 28 strains with BSHs paralogs in 120 strains, the BSHs from *Bacteroides*, *Clostridium*, *Lactobacillus*, *Ruminococcus*, and *Marvinbryantia* showed varied phylotype distributions. Overall, nine strains with paralogs were distributed in the same phylotype (Additional file 1: Figure S8, marked by black triangles), 15 strains were distributed in two phylotypes (Additional file 1: Figure S8, marked by red triangles). Specifically, most of the strain with paralogs were from *Bacteroides*, and mainly distributed in two phylotypes, i.e., BSH-T5 and BSH-T6 (right panel of Fig. 4, Additional file 1: Figure S8). Four strains were distributed both within and between phylotypes (Additional file 1: Figure S8). Thus, different paralogs of BSHs within a genus could have sequence dissimilarity, which might lead to variable functional roles of these two genera in bile acid metabolism.

### Molecular docking and enzyme activity comparisons between eight BSH phylotypes

**Fig. 5:**
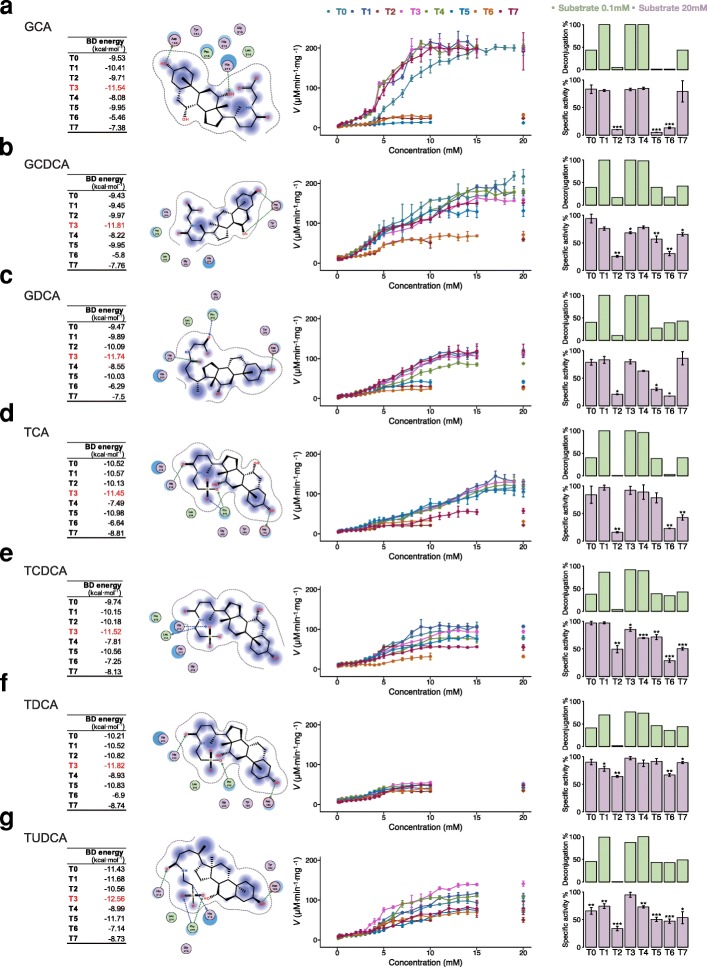
Molecular docking and enzyme activity comparison. The results of molecular docking and kinetic reaction of eight BSH phylotypes with **a** glycocholic acid (GCA), **b** glycochenodeoxycholic acid (GCDCA), **c** glycine deoxycholic acid (GDCA), **d** taurocholic acid (TCA), **e** taurochenodeoxycholic acid (TCDCA), **f** taurodeoxycholic acid (TDCA), and **g** tauroursodeoxycholic acid (TUDCA). The left panel shows the binding energy (BD energy) of each BSH, and molecular docking predicted binding models of BSH-T3 with different bile acids. The middle panel showed the eight BSHs in the kinetic reaction with different bile acids. The right panel compares the deconjugation (the proportion of product bile acid of specific substrate) in light green bar when the substrate concentration is 0.1 mM, and specific activity (the activity of an enzyme per milligram of total protein, using the highest enzyme activity as 100%) of eight BSHs with different bile salts (final concentration of all bile salts was 20 mM) in light purple bar when the substrate concentration is 20 mM. Data were analyzed with student’s *t* test corrected by false discovery rate. *p < 0.05, **p < 0.01, and ***p < 0.05, versus the BSH which shown the highest enzyme activity for the specific bile salt, i.e., BSH-T3 showed the highest enzyme activity when the substrates were TCDCA and TDCA, for TCA, it is BSH-T1. Details are shown in Additional file 2: Table S7–S9

To compare the hydrolysis capacity among the eight phylotypes of BSH, BSHs with the highest RA in each phylotype were selected for molecular docking and enzyme activity assays (Additional file 2: Table S7). A lower binding energy (BD energy) of molecular docking usually indicated more stability of the complex, and the residues of eight representative BSH contact to seven substrates were extracted from the complex (Additional file 1: Figure S10-S17). From the results, BSH-T3 showed the most stable complex, containing all seven bile acids, and the residues of BSH-T3 contacted with substrates were Asp183, His212, His213, Leu214, and Pro215 (left panel of Fig. 5, Additional file 1: Figure S13).

Representative BSHs of the eight phylotypes were synthesized and purified (Additional file 1: Figure S9a) as described in the “Methods” section. The deconjugation of BSH at lowest concentration (0.1 mM) of substrates was confirmed by LC-MS/MS (right panel of Fig. 5, Additional file 2: Table S9). The results of enzyme activity assay shown that BSH-T0, BSH-T1, BSH-T3, BSH-T4, and BSH-T7 performed higher specific activity (the activity of an enzyme per milligram of total protein, using the highest enzyme activity as 100% for each substrate) among seven bile salts (middle and right panel of Fig. 5, Additional file 2: Table S8). However, different BSH phylotypes display selective deconjugation activity based on substrates. The specific activity of BSH-T5 was lower when the substrate was glycocholic acid (GCA) and both GCA and taurocholic acid (TCA) for BSH-T6 (middle and right panel of Fig. 5, Additional file 1: Figure S18). In particular, BSH-T1, BSH-T3, and BSH-T4 showed the highest specific activity with GCA, while BSH-T0 and BSH-T2 showed the highest specific activity with glycochenodeoxycholic acid (GCDCA), and BSH-T0 showed the highest specific activity when taurochenodeoxycholic acid (TCDCA) was the substrate (Additional file 1: Figure S18). It is worth noting that BSH-T1, which had the highest abundance of BSHs in the human gut microbiota, exhibited higher deconjugation activity (middle and right panel of Fig. 5). BSH-T2 showed lower enzyme activity both in silico and in vitro (Fig. 5, Additional file 1: Figure S12, S18). Comparatively, BSH-T7 showed higher deconjugation activity in vitro but not in silico (middle and right panel of Fig. 5, Additional file 1: Figure S17, S18). This discrepancy was likely because the computational molecular docking only partially reflected the actual enzyme activities. Nevertheless, computational work is helpful to understand the biological activity at the molecular level.

### Variation in patterns of BSHs in health/disease cohorts

To further investigate the functional implications of BSH, we analyzed the relationship between the RA of BSHs and disease status of different groups, including populations of geographical regions and health/disease cohorts.

First, we analyzed the relationship between the RA of BSHs in our target populations with the World Health Organization (WHO) released phenotypes, namely, death rate of diabetes, death rate of cardiovascular diseases (CVD), mean blood cholesterol, and BMI of obesity (Additional file 1: Figure S19, Additional file 2: Table S10). We found that the RA of all BSH was significantly correlated with death rate of diabetes (*r* = − 0.65, *p* = 0.03) (Additional file 1: Figure S19a, entry 7 of Additional file 1: Figure S19c), and the RA of all BSH-T0 was significantly correlated with death rate of CVD (*r* = 0.92, *p* = 0.0041) (Additional file 1: Figure S19b, entry 7 of Additional file 1: Figure S19c). These findings might indicate that the RA of BHSs was relevant to CVD risk among different populations. However, it should be noted that the metagenome data and epidemiological data did not originate from the same cohort in this association study, which may be a limitation of this investigation. Nevertheless, these results still shed light on the relationship of BSH with various diseases.

**Fig. 6:**
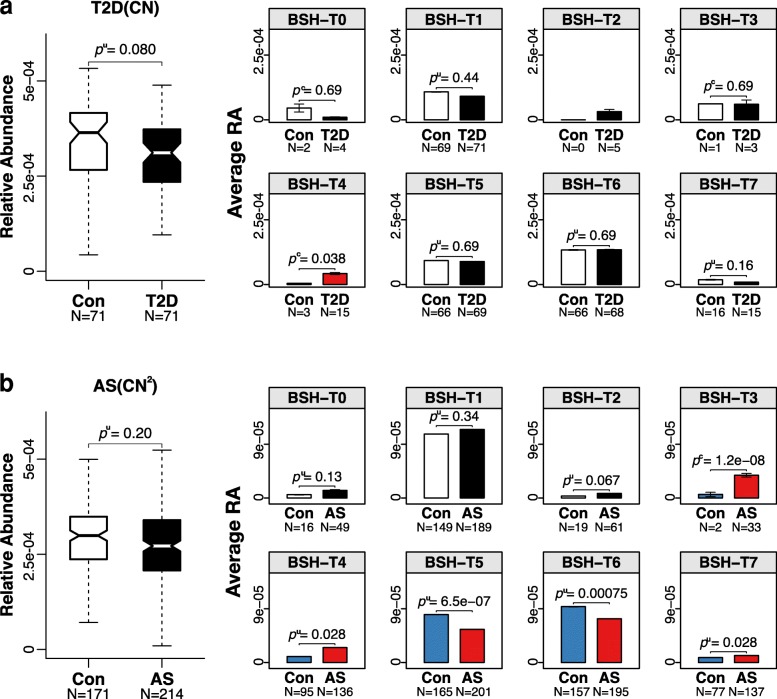
The relative abundance of BSHs among case-control cohorts. **a** The relative abundance (RA) of BSH in the gut microbiome of healthy people and patients with type 2 diabetes (T2D) and the average RA of different BSH phylotypes in healthy people and patients. **b** The relative abundance (RA) of BSH in the gut microbiome of healthy people and patients with atherosclerosis (AS) and the average RA of different BSH phylotypes in healthy people and patients. *p*^c^ values were calculated by the chi-squared test, *p*^*u*^ values were calculated by the Mann-Whitney *U* test, and *p* values in each type were followed by false discovery rate correction. The populations from CN and CN^2^ are independent cases. Details are shown in Additional file 2: Table S3

The gut metagenome data of the 11 worldwide populations presented above were extracted from the control group of various cohort studies as described in the “Methods” section. Therefore, we further explored the variation patterns of BSHs among health/disease cohorts (Fig. 6). In accordance with the population-level results, the RA of BSH showed no significant differences between healthy individuals and colorectal cancer (CRC), adenoma, impaired glucose tolerance (IGT), or type 2 diabetes (T2D) patients other than Chinese (Additional file 1: Figure S20). However, the RA of BSH-T4 in Chinese with T2D was significantly higher than that of healthy individuals (*p*^*u*^ = 0.038, Fig. 6a). Meanwhile, the RA of BSH-T3, BSH-T4, and BSH-T7 in populations with atherosclerosis (AS) were significantly higher (BSH-T3: *p*^*c*^ = 1.2e-8; BSH-T4: *p*^*u*^ = 0.028; BSH-T7: *p*^*u*^ = 0.028), whereas the RA of BSH-T5 and BSH-T6 were significantly lower (BSH-T5: *p*^*u*^ = 6.5e-7; BSH-T6: *p*^*u*^ = 0.00075) than that of healthy individuals (Fig. 6b). These results indicated that BSH maybe had pleiotropic impact on the development and progression of AS. However, additional studies are required to derive a reasonable relationship between bile acid metabolism and various diseases.

## Discussion

In our study, BSH protein sequences (BSHs) were used for BLASTP searches to determine their sensitivity relative to other gene sequences. The average sequence identity of BSHs between genera was 44.46%, while it was 82.13% within genera (Additional file 1: Figure S3b). Thus, the BLOSUM45 matrix and cutoff of 45% homology were used build reference BSHs while ensuring all BSHs would be screened from the HMP database. Additionally, the BLOSUM62 with parameters of e-value 1e-5 and an identity of 62% as the cutoff were used to identify BSHs sequences in each individual to the accurate taxonomic assignments of BSHs in the human gut microbiota.

To the best of our knowledge, there have been few studies of paralogs of the BSH gene in bacterial genomes. Based on our results, the taxonomic characteristics of reference BSHs showed that 27.52% of the BSHs encoding bacteria behaved as paralogs (Fig. 2d). The presence of paralogs may cause differences in the BSHs and their functions in the same bacteria strain. Thus, we reclassified the 156 BSHs to eight phylotypes based on the phylogenetic tree, and the taxonomic characteristics of different phylotypes were diverse. Interestingly, most of paralogs between phylotypes were belonging to BSH-T5 and BSH-T6 and distributed in *Bacteroides* (Additional file 1: Figure S8). At the bacterial level, Yao et al. performed a screen of the BSH activity of 20 *Bacteroidetes* strains found in the human gut, and these majority strains display some degree of selectivity for conjugated bile acid substrates [35]. At the genetic level, our results have demonstrated that different BSHs in the same *Bacteroides* strain also have differences in deconjugation ability. Moreover, the BSHs from the same strain but belonged to different phylotypes, i.e., representative BSHs of BSH-T5 and BSH-T6 from *Bacteroides uniformis* ATCC 8492, could also exhibit different deconjugation ability (Fig. 5).

Because of their importance in human metabolism, there have been several studies investigating BSH. However, most previous studies focused on cloning BSH from limited bacteria, such as *Bacteroidetes* strains [35], *Lactobacillus* [28, 36–39], *Bifidobacterium* [40–42], *Listeria* [43, 44], *Enterococcus* [45, 46], and *Clostridium* [17]. Fang et al. revealed the complexity of bile resistance level determination in commensal *L. salivarius* strains [28]. Sun et al. presented a robust phylogenomic framework of existing species and for classifying new species of *Lactobacillus* [47]. Liang et al. divided BSH sequences from *Lactobacillaceae* into five groups based on phylogenetic analysis [48], while Jones et al. divided BSH genes identified in fecal microbiota of 15 humans into three clusters and performed a functional analysis of BSH activity [49]. The present study benefited from the use of next-generation sequencing technology and the abundance of public metagenomic databases, enabling a comprehensive investigation of BSH. First, we presented the taxonomic characteristics of BSHs in the human gut microbiome as widely and comprehensively as possible. Then, we proposed a new classification framework of BSHs based on phylogenetic relationship and functionally separated and characterized them both in silico and in vitro. Moreover, we disclosed the relationship between the abundance of BSHs and diseases from public gut metagenome cohort data. As a result, the present study of BSH is more comprehensive than prior studies and provides a phylogenetic-based system to evaluate BSHs activity by classifying the target sequence into specific phylotype.

Several previous studies have reported that changes in bile acids are closely connected with managing blood cholesterol level [4], diabetes [50–54], obesity [55], inflammatory bowel disease (IBD) [56], Crohn’s disease [57], and cardiovascular diseases (CVD) [5, 58–63]. Furthermore, Labbé A et al. investigated the change of bile acid modification genes from IBD and T2D patients with bacterial taxonomic analysis in the gut microbiome [52]. The reported association between bile acid and various diseases is pleiotropic, and the extremely low [51] or high [6] bile acid may both be related to some disease. In this paper, we investigated the relationship between relative abundance (RA) of BSHs and above-mentioned diseases. Specifically, the RA of the phylotype with the highest BSH activity (BSH-T3) was higher in patients with AS, while the RA of the phylotype with intermediate activity (BSH-T5, T6) was higher in healthy people (Fig. 6). The results suggested that BSH usually displayed beneficial effect on metabolism, but the higher RA of high activity BSH phylotype may have adverse effects and promoting the occurrence and development of diseases. It also demonstrates the importance of BSH classification, i.e., exploring the relationship of RA of BSH and disease should firstly distinguish specific BSH with activity assessment rather than using rough RA of total BSHs. Taken together, BSH indeed plays a role in some diseases, but it could be pleiotropic signals, causal analysis of the relationship between BSH and bile acid metabolism-related phenotypes still requires subsequent biological studies in the future.

It should be noted that this study has several limitations: (1) the data quality among different metagenome databases is not consistent (e.g., sample sizes, sequencing depth, individuals selected, and standards differed); (2) the metagenome data lack individual dietary and other information such as data describing climate, local habitats, and lifestyle; (3) the individuals described by the metagenome data and epidemiological data of the WHO were not directly related in the populational-level correlation analysis; (4) the results of BSH activity comparison in silico and in vitro were not exactly identical; and (5) the in vivo effects of different BSHs on various bile acids were not evaluated. Despite the limitations, the results are still valid for two reasons: the activity of different BSHs is evaluated by substantial enzyme kinetics and confirmed by LC-MS/MS approach; the relationship of BSHs and diseases is assessed by both populational association and cohort study with big sample sizes.

## Conclusions

In this study, we describe the distribution of bacteria that expressed BSH and discuss their distribution and abundance in worldwide populations. Based on the above series of analyses, we propose a new method to reclassify the BSHs and compare the enzyme activities between phylotypes. Moreover, we found the RA of some BSH phylotypes that are significantly correlated with T2D and AS, but the effects are pleiotropic, which highlights the importance of BSH classification in the future studies of BA metabolism-related diseases.

## Methods

### Sequences and structures comparison

The known 3D structures of BSHs were searched in the protein data bank (PDB) [25] using the keyword “bile salt hydrolase” and “Choloylglycine hydrolase”. Only 12 of the 25 searched results are our purpose structures (Additional file 1: Figure S2b). Pairwise amino acid sequence alignments of the BSHs were performed by BLASTP (v 2.2.29+) [64], and multiple alignments were conducted using DNAMAN (v 8.0). The secondary structures of these BSHs were downloaded from PDBsum [65]. Comparison of 3D structures was conducted using MOE (v 2014).

### Taxonomic characterization

The reference genomes of 1751 bacterial strains covering 1253 species were obtained from the HMP database [32] in September 2014. The genes and related proteins from these bacterial genomes were predicted by MetaGeneMark (v 2.8) [66], and the taxonomic information regarding these genes and proteins was directly extracted from the strain names.

The query BSH protein sequences were collected from the Refseq database of NCBI database [24] using the keyword “bile salt hydrolase” and “Choloylglycine hydrolase”. Then, the total number of amino acids in the sequences was limited between 300 and 400. The sequence identity interval of the BSHs between and within genera was calculated from these 765 sequences to define the thresholds. Thus, the final 591 BSHs reference sequences (screening was performed by controlling the total length of the sequences from 300 to 400) were identified from HMP database by taking the initial 765 BSH as query and using BLASTP with e-value of 1e-5 and sequence identity of 45% as cutoff (Additional file 1: Figure S2a).

### Publicly available metagenomic sequence data

The metagenomic sequence data of individuals were collected from 11 populations of six continents, including the Hadza ethnic group of Tanzania (HZ) of Africa [33, 67], China (CN) of Asia [50, 68], Japan (JP) of Asia [69], South Korea (KR) of Asia [70], Denmark (DK) of Europe [71], Sweden (SE) of Europe [72], Austria (AT) of Europe [56], France (FR) of Europe [73], Australia (AU, PRJEB6092) of Oceania, the United States (US) of North America (N-America) [21, 32], and Peru (PE) of South America (S-America) [74].

To construct metagenomic datasets of healthy individuals from each country, we screened out the data for individuals with 18.5 < BMI < 29.9 kg/m^2^ [69, 75], 12 < age < 75 [69, 76], and those designated with diseases that were excluded from the data. Although we could not access the metadata for individuals from the United States, we used all data from healthy individuals with an average BMI of 24 ± 4 kg/m^2^ for this cohort. Finally, a total of 581 healthy individuals were selected from these 11 countries for analysis (Additional file 2: Table S3).

To study the differences in the BSH relative abundance (RA) between healthy individuals and patients, we used metagenomic datasets of individuals from different countries who were identified with diseases expected to be related to deviations in bile acids such as colorectal cancer (CRC), adenoma, type 2 diabetes (T2D), impaired glucose tolerance (IGT), and atherosclerosis (AS) [58] (Additional file 2: Table S3).

### Metagenomic analysis based on different populations

All raw sequencing reads were assessed and filtered using the FASTX-Toolkit, and the high quality microbiome sequencing reads were assembled with the SOAPdenovo2 (v 2.04) [77] package. After assembly, contigs with at least 500 bp were further used to predict the genes with MetaGeneMark (v 2.8), after which a non-redundant protein set was constructed by pair-wise comparison of all protein sequences within populations using BLASTP (v 35x1) [78] with 95% identity and 90% overlapping thresholds. The relative abundance (RA) of each protein sequence in each individual was calculated based on the number of read pairs mapped to the gene over the length of the protein sequence and divided by the summary of sequence abundance per individual [50]. The cumulative RA was calculated by the sum of RA of each genus in each population. The BSHs were identified in the above population database using BLASTP with an e-value = 1e-5 and 62% sequence identity as the cutoff values.

### Phylogenetic tree

A phylogenetic tree was built using the maximum likelihood method in the MEGA software (v 7.0). Dendroscope (v 3.4.7) was used to embellish the phylogenetic tree by adjusting the labels and filling the colors as needed.

### Related physiological/diseases data

Nationwide blood cholesterol (2008), obese BMI (2014), diabetes death rate (per 100,000 individuals; 2012) and cardiovascular diseases death rate (per 100,000 individuals; 2012) data by country were downloaded from the World Health Organization (WHO) [79] (Additional file 2: Table S10).

### Homology modeling and molecular docking

The online software, Protein Homology/analogY Recognition Engine (Phyre2, V 2.04) [80], was employed to predict the homologous structure of BSHs using intensive mode [81]. Ligands including glycocholic acid (GCA, C_26_H_43_NO_6_), glycochenodeoxycholic acid (GCDCA, C_26_H_43_NO_5_), glycine deoxycholic acid (GDCA, C_26_H_43_NO_5_), taurocholic acid (TCA, C_26_H_45_NO_7_S), taurochenodeoxycholic acid (TCDCA, C_26_H_45_NO_6_S), taurodeoxycholic acid (TDCA, C_26_H_45_NO_6_S), and tauroursodeoxycholic acid (TUDCA, C_26_H_45_NO_6_S) were obtained from the ZINC database [82]. The AutoDock program (v 4.2.6) [83] was employed to generate an ensemble of docked conformations for each ligand bound to its target. Four units of BSH were found to bond with single bile acid substrate molecules by forming a tetramer. We then used the genetic algorithm for conformation searches and conducted 100 individual genetic algorithm (GA) runs to generate 100 docked conformations for each ligand.

### Materials, bacterial strains, and vectors

Sodium glycocholate hydrate (CAS: 863-57-0), sodium glycochenodeoxycholate (CAS: 16564-43-5), sodium glycodeoxycholate (CAS: 16409-34-0), sodium taurocholate hydrate (CAS: 345909-26-4), sodium taurochnodeoxycholate (CAS: 6009-98-9), sodium taurodeoxycholate hydrate (CAS: 207737-97-1), sodium tauroursodeoxycholate (CAS: 35807-85-3), CA-d4 (CAS: 116380-66-6), DCA-d4 (CAS: 112076-61-6), CDCA-d4 (CAS: 99102-69-9), UDCA-d4 (CAS: 347841-46-7), glycine (CAS: 56-40-6), and taurine (CAS: 107-35-7) were used in the enzyme assay. pET28a (+) was used for the expression of His-tagged (6x) recombinant BSH (Genscript, Nanjing, China). The target BSH sequences were ligated into the NcoI/XhoI-digested vector pET28a (+) (T7 promoter), resulting in six recombinant plasmid pET28a (+)-*bsh* genes being transformed into *E. coli* BL21 (DE3) competent cells.

### Expression and purification of BSH

The eight *bsh*-recombinant bacteria were inoculated into Luria-Bertani (LB) broth containing 50 μg·ml^−1^ kanamycin, and expression of the BSH genes was induced by the addition of isopropyl β-D-thiogalactoside (IPTG, 0.5 mM). The cell pellet obtained by harvesting was subsequently resuspended (1:10) in buffer solution (0.05 M Tris, 0.05 M NaCl, 0.5 mM EDTA, 5% Glycerol, pH 7.9) and disrupted by sonication (alternating pulses: on for 3 s, off for 3 s; 60% amplitude) until clear solutions were obtained.

The BSH was purified by nickel-nitrilotriacetic acid (Ni^2+^-NTA) agarose column (HisTrap™ HP, GE Healthcare, USA). The presence and purity of BSH in each sample were confirmed by SDS-PAGE. After which pure BSH proteins were stored at − 80 °C after subsequently lyophilized.

### Kinetics of BSH reaction

The BSH specific activity was determined by measuring the release of amino acids (glycine and taurine) from conjugated bile salts. The amounts of amino acids liberated by BSH were determined by ninhydrin assay [84]. Protein concentrations were determined using a Bradford Protein Assay kit (Beyotime, Nanjing, China). The purified BSH-lyophilized powders were diluted with 20 mM phosphate buffer (pH 6.5) until the concentration of BSH was 0.65 mg·ml^−1^. Next, 10 μl of BSH liquid, 10 μl of bile salts, and 180 μl of reaction buffer (20 mM phosphate buffer, pH 6.5) were mixed, and 10 μl of liquid paraffin were added. The samples were subsequently incubated at 37 °C for 30 min, after which used 200 μl of 15% (*w*/*v*) trichloroacetic acid to terminate the reaction, and the sample was centrifuged to remove the precipitates. Mix 10 μl of reaction supernatant with 190 μl of ninhydrin reagent and kept in a boiling water bath for 15 min. The absorbance at 570 nm was measured in a 96-well plate. A standard BSH activity curve was subsequently prepared for glycine and taurine (Additional file 1: Figure S11b). One unit of BSH activity was defined as the amount of enzyme that released 1 μmol of amino acid from the substrate per min.

### LC-MS/MS assay

Analysis was performed on a triple quadrupole tandem liquid chromatography-mass spectrometry (LC-MS/MS) system (LCMS8050, Shimadzu). Chromatographic separation was performed on an ACQUITY UPLC BEH C18 (2.1 × 100 mm, 1.7 μm) column. The mobile phase consisted of 0.1% formic acid (mobile phase A) in water and 0.1% formic acid in acetonitrile (mobile phase B) running at a flow rate of 0.4 ml/min. The gradient elution program was 5% B at 0–1 min, 5–42% at 1–2.5 min, 42–45% at 2.5–5.5 min, 45–60% at 5.5–8 min, 60–95% at 8-9 min, 95% at 9–9.5 min, back to initial conditions, and 2 min for equilibration. The column was maintained at 55 °C and the injection volume of each sample was 1 μl.

The LC-MS/MS system control and data analyses were performed by LabSolutions software (the software version: version 5.65). The ion source parameters were set as follows: nebulizing gas flow of 2 l/min, heating gas flow of 10 l/min, interface temperature of 300 °C, DL temperature of 250 °C, heat block temperature of 400 °C, and drying flow of 10 l/min. The data were collected with multiple reaction monitor (MRM) in negative mode.

An isotope-labeled standard calibration approach with standard addition was used to avoid the matrix effects and ensure the accuracy of measurement. Calibration curves were constructed each day using seven calibrators prepared from pooled plasma spiked with CA, DCA, CDCA, and UDCA at concentration range of 0.00625–0.2 mM and CA-d4, DCA-d4, CDCA-d4, and UDCA-d4 at 0.003125 mM (Additional file 1: Figure S21, S22).

### Statistical analysis

All values were expressed as mean ± SEM. Statistically significant differences between two groups were determined by Mann-Whitney *U* test followed by false discovery rate correction. The relation analysis was done with the Spearman rank correlations. Multiple comparisons were performed by multivariable-adjusted analysis using linear regression model. All analyses were performed using R (v 3.3.2), and *p* < 0.05 was considered as statistically significant.

## Additional files

Additional file 1: Figure S1. The enterohepatic circulation of bile acids. Figure S2. The analysis flowcharts. Figure S3. Genera characterization of BSHs obtained from NCBI database. Figure S4. The sequence identity of reported BSH sequences. Figure S5. Reported conserved residues of BSH in PDB database. Figure S6. Taxonomic identification of BSHs in HMP database. Figure S7. The Taxonomic identification and paralogs distribution of BSHs in HMP. Figure S8. Phylogenetic tree of BSHs in the gut microbiome of 11 populations. Figure S9. SDS-PAGE of BSH and Standard curve used in ninhydrin assay. Figure S10. Molecular docking results between BSH-T0 and bile acids. Figure S11. Molecular docking studies between BSH-T1 and bile acids. Figure S12. Molecular docking studies between BSH-T2 and bile acids. Figure S13. Molecular docking studies between BSH-T3 and bile acids. Figure S14. Molecular docking studies between BSH-T4 and bile acids. Figure S15. Molecular docking studies between BSH-T5 and bile acids. Figure S16. Molecular docking studies between BSH-T6 and bile acids. Figure S17. Molecular docking studies between BSH-T7 and bile acids. Figure S18. Enzyme activity comparisons between substrates in each BSH phylotype. Figure S19. Relationship between average RA of BSHs with WHO released phenotypes in 11 populations. Figure S20. The relative abundance of BSHs among case-control cohorts. Figure S21. Standard curve of bile acids used in LC-MS/MS. Figure S22. Typical LC spectrum of different bile acids. (PDF 4413 kb)

Additional file 2: Table S1. Reported conserved residues of BSH in PDB database. Table S2. Taxonomic identification of BSHs in HMP database. Table S3. The information of individuals from the 11 populations. Table S4. Cumulative relative abundance of reclassified BSHs in 11 populations. Table S5. Cumulative relative abundance of BSHs in different genera in 11 populations. Table S6. The percentage of relative abundance of each BSH phylotype and each population. Table S7. Information of represent BSHs used in molecular docking and enzyme activity assay. Table S8. BSH activity at a substrate concentration of 20 mM by ninhydrin assay. Table S9. Percent deconjugation of BSH at a substrate concentration of 0.1 mM. Table S10. Information of WHO released phenotypes in 11 populations. (XLSX 100 kb)

## Abbreviations

AS: Atherosclerotic
AT: Austria
AU: Australia
BAs: Bile acids
BD Energy: Binding energy
BSH: Bile salt hydrolase
BSHs: BSH protein sequences
CN: China
CRC: Colorectal cancer
CVD: Cardiovascular disease
DK: Denmark
FR: France
GA: Genetic algorithm
GCA: Glycocholic acid
GCDCA: Glycochenodeoxycholic acid
GDCA: Glycine deoxycholic acid
HMP: Human Microbiome Project
HZ: Hadza ethnic group of Tanzania
IGT: Impaired glucose tolerance
IPTG: Isopropyl β-D-thiogalactoside
JP: Japan
KR: South Korea
MetaHIT: META genomics of the Human Intestinal Tract
N-America: North America
PE: Peru
RA: Relative abundance
S-America: South America
SE: Sweden
T2D: Type 2 diabetes
TCA: Taurocholic acid
TCDCA: Taurochenodeoxycholic acid
TDCA: Taurodeoxycholic acid
TLDCA: Taurolithocholic acid
TUDCA: Tauroursodeoxycholic acid
US: The United States
WHO: World Health Organization

## References

[1] Wang HH, Portincasa P, Wang DQ (2008). Molecular pathophysiology and physical chemistry of cholesterol gallstones. Front Biosci.

[2] Copaci I, Micu L, Iliescu L, Voiculescu M (2005). New therapeutical indications of ursodeoxycholic acid. Rom J Gastroenterol.

[3] Cariou B, Chetiveaux M, Zair Y, Pouteau E, Disse E, Guyomarc’h-Delasalle B (2011). Fasting plasma chenodeoxycholic acid and cholic acid concentrations are inversely correlated with insulin sensitivity in adults. Nutr Metab (Lond).

[4] Joyce SA, MacSharry J, Casey PG, Kinsella M, Murphy EF, Shanahan F (2014). Regulation of host weight gain and lipid metabolism by bacterial bile acid modification in the gut. Proc Natl Acad Sci U S A.

[5] Charach G, Argov O, Geiger K, Charach L, Rogowski O, Grosskopf I (2017). Diminished bile acids excretion is a risk factor for coronary artery disease: 20-year follow up and long-term outcome. Ther Adv Gastroenterol.

[6] Ignacio Barrasa J, Olmo N, Perez-Ramos P, Santiago-Gomez A, Lecona E, Turnay J (2011). Deoxycholic and chenodeoxycholic bile acids induce apoptosis via oxidative stress in human colon adenocarcinoma cells. Apoptosis.

[7] Parker HE, Wallis K, le Roux CW, Wong KY, Reimann F, Gribble FM (2012). Molecular mechanisms underlying bile acid-stimulated glucagon-like peptide-1 secretion. Br J Pharmacol.

[8] Studer E, Zhou X, Zhao R, Wang Y, Takabe K, Nagahashi M (2012). Conjugated bile acids activate the sphingosine-1-phosphate receptor 2 in primary rodent hepatocytes. Hepatology.

[9] Degirolamo C, Rainaldi S, Bovenga F, Murzilli S, Moschetta A (2014). Microbiota modification with probiotics induces hepatic bile acid synthesis via downregulation of the Fxr-Fgf15 axis in mice. Cell Rep.

[10] Makishima M, Okamoto AY, Repa JJ, Tu H, Learned RM, Luk A (1999). Identification of a nuclear receptor for bile acids. Science.

[11] Haeusler RA, Astiarraga B, Camastra S, Accili D, Ferrannini E (2013). Human insulin resistance is associated with increased plasma levels of 12alpha-hydroxylated bile acids. Diabetes.

[12] Cipriani S, Mencarelli A, Chini MG, Distrutti E, Renga B, Bifulco G (2011). The bile acid receptor GPBAR-1 (TGR5) modulates integrity of intestinal barrier and immune response to experimental colitis. PLoS One.

[13] Wang Z, Zeng X, Mo Y, Smith K, Guo Y, Lin J (2012). Identification and characterization of a bile salt hydrolase from *Lactobacillus salivarius* for development of novel alternatives to antibiotic growth promoters. Appl Environ Microbiol.

[14] Corzo G, Gilliland SE (1999). Bile salt hydrolase activity of three strains of *Lactobacillus acidophilus*. J Dairy Sci.

[15] Kim GB, Miyamoto CM, Meighen EA, Lee BH (2004). Cloning and characterization of the bile salt hydrolase genes (*bsh*) from *Bifidobacterium bifidum* strains. Appl Environ Microbiol.

[16] De Filippo C, Cavalieri D, Di Paola M, Ramazzotti M, Poullet JB, Massart S (2010). Impact of diet in shaping gut microbiota revealed by a comparative study in children from Europe and rural Africa. Proc Natl Acad Sci U S A.

[17] Coleman JP, Hudson LL (1995). Cloning and characterization of a conjugated bile acid hydrolase gene from *Clostridium perfringens*. Appl Environ Microbiol.

[18] Stellwag EJ, Hylemon PB (1976). Purification and characterization of bile salt hydrolase from *Bacteroides fragilis* subsp. *fragilis*. Biochim Biophys Acta.

[19] Chae JP, Valeriano VD, Kim GB, Kang DK (2013). Molecular cloning, characterization and comparison of bile salt hydrolases from *Lactobacillus johnsonii* PF01. J Appl Microbiol.

[20] Bi J, Fang F, Lu S, Du G, Chen J (2013). New insight into the catalytic properties of bile salt hydrolase. J Mol Catal B Enzym.

[21] Human Microbiome Project C (2012). Structure, function and diversity of the healthy human microbiome. Nature.

[22] Li J, Jia H, Cai X, Zhong H, Feng Q, Sunagawa S (2014). An integrated catalog of reference genes in the human gut microbiome. Nat Biotechnol.

[23] Liu W, Zhang J, Wu C, Cai S, Huang W, Chen J (2016). Unique features of ethnic Mongolian gut microbiome revealed by metagenomic analysis. Sci Rep.

[24] National Center for Biotechnology Information, U.S. https://www.ncbi.nlm.nih.gov. Accessed 17 Oct 2018.

[25] RCSB: Protein Data Bank. https://www.rcsb.org. Accessed 20 Oct 2018.

[26] Rossocha M, Schultz-Heienbrok R, von Moeller H, Coleman JP, Saenger W (2005). Conjugated bile acid hydrolase is a tetrameric N-terminal thiol hydrolase with specific recognition of its cholyl but not of its tauryl product. Biochemistry.

[27] Kumar RS, Brannigan JA, Prabhune AA, Pundle AV, Dodson GG, Dodson EJ (2006). Structural and functional analysis of a conjugated bile salt hydrolase from *Bifidobacterium longum* reveals an evolutionary relationship with penicillin V acylase. J Biol Chem.

[28] Fang F, Li Y, Bumann M, Raftis EJ, Casey PG, Cooney JC (2009). Allelic variation of bile salt hydrolase genes in *Lactobacillus salivarius* does not determine bile resistance levels. J Bacteriol.

[29] Chand D, Panigrahi P, Varshney N, Ramasamy S, Suresh CG (2018). Structure and function of a highly active bile salt hydrolase (BSH) from *Enterococcus faecalis* and post-translational processing of BSH enzymes. Biochim Biophys Acta, Proteins Proteomics.

[30] Geng W, Lin J (2016). Bacterial bile salt hydrolase: an intestinal microbiome target for enhanced animal health. Anim Health Res Rev.

[31] Oinonen C, Rouvinen J (2000). Structural comparison of Ntn-hydrolases. Protein Sci.

[32] Human Microbiome Project. https://www.hmpdacc.org. Accessed 24 Sept 2014.

[33] Schnorr SL, Candela M, Rampelli S, Centanni M, Consolandi C, Basaglia G (2014). Gut microbiome of the Hadza hunter-gatherers. Nat Commun.

[34] Mancabelli L, Milani C, Lugli GA, Turroni F, Ferrario C, van Sinderen D (2017). Meta-analysis of the human gut microbiome from urbanized and pre-agricultural populations. Environ Microbiol.

[35] Yao L, Seaton SC, Ndousse-Fetter S, Adhikari AA, DiBenedetto N, Mina AI, et al. A selective gut bacterial bile salt hydrolase alters host metabolism. Elife. 2018;7:e37182. 10.7554/eLife.37182 PMC6078496 30014852

[36] Lambert JM, Bongers RS, de Vos WM, Kleerebezem M (2008). Functional analysis of four bile salt hydrolase and penicillin acylase family members in *Lactobacillus plantarum* WCFS1. Appl Environ Microbiol.

[37] Dong Z, Zhang J, Li H, Du G, Chen J, Lee B (2015). Codon and propeptide optimizations to improve the food-grade expression of bile salt hydrolase in Lactococcus lactis. Protein Pept Lett.

[38] Allain T, Chaouch S, Thomas M, Travers MA, Valle I, Langella P (2018). Bile salt hydrolase activities: a novel target to screen anti-*giardia* Lactobacilli?. Front Microbiol.

[39] Corzo G, Gilliland SE (1999). Measurement of bile salt hydrolase activity from *Lactobacillus acidophilus* based on disappearance of conjugated bile salts. J Dairy Sci.

[40] Kim GB, Brochet M, Lee BH (2005). Cloning and characterization of a bile salt hydrolase (*bsh*) from *Bifidobacterium adolescentis*. Biotechnol Lett.

[41] Kumar RS, Brannigan JA, Pundle A, Prabhune A, Dodson GG, Suresh CG (2004). Expression, purification, crystallization and preliminary X-ray diffraction analysis of conjugated bile salt hydrolase from *Bifidobacterium longum*. Acta Crystallogr D Biol Crystallogr.

[42] Tanaka H, Hashiba H, Kok J, Mierau I (2000). Bile salt hydrolase of *Bifidobacterium longum*-biochemical and genetic characterization. Appl Environ Microbiol.

[43] Dussurget O, Cabanes D, Dehoux P, Lecuit M, Buchrieser C, Glaser P (2002). *Listeria monocytogenes* bile salt hydrolase is a PrfA-regulated virulence factor involved in the intestinal and hepatic phases of listeriosis. Mol Microbiol.

[44] Begley M, Sleator RD, Gahan CG, Hill C (2005). Contribution of three bile-associated loci, bsh, pva, and btlB, to gastrointestinal persistence and bile tolerance of *Listeria monocytogenes*. Infect Immun.

[45] Wijaya A, Hermann A, Abriouel H, Specht I, Yousif NM, Holzapfel WH (2004). Cloning of the bile salt hydrolase (*bsh*) gene from *Enterococcus faecium* FAIR-E 345 and chromosomal location of bsh genes in food Enterococci. J Food Prot.

[46] Franz CMAP, Specht I, Haberer P, Holzapfel WH (2001). Bile salt hydrolase activity of enterococci isolated from food: screening and quantitative determination. J Food Prot.

[47] Sun Z, Harris HM, McCann A, Guo C, Argimon S, Zhang W (2015). Expanding the biotechnology potential of lactobacilli through comparative genomics of 213 strains and associated genera. Nat Commun.

[48] Liang L, Yi Y, Lv Y, Qian J, Lei X, Zhang G. A comprehensive genome survey provides novel insights into bile salt hydrolase (BSH) in *Lactobacillaceae*. Molecules. 2018;23(5):1157. 10.3390/molecules23051157 PMC6100381 29751655

[49] Jones BV, Begley M, Hill C, Gahan CG, Marchesi JR (2008). Functional and comparative metagenomic analysis of bile salt hydrolase activity in the human gut microbiome. Proc Natl Acad Sci U S A.

[50] Qin J, Li Y, Cai Z, Li S, Zhu J, Zhang F (2012). A metagenome-wide association study of gut microbiota in type 2 diabetes. Nature.

[51] Steiner C, Othman A, Saely CH, Rein P, Drexel H, von Eckardstein A, et al. Bile acid metabolites in serum: Intraindividual variation and associations with coronary heart disease, metabolic syndrome and diabetes mellitus. PLoS One. 2011;6(11):e25006. 10.1371/journal.pone.0025006 PMC3215718 22110577

[52] Suhre K, Meisinger C, Doring A, Altmaier E, Belcredi P, Gieger C (2010). Metabolic footprint of diabetes: a multiplatform metabolomics study in an epidemiological setting. PLoS One.

[53] Haeusler RA, Astiarraga B, Camastra S, Accili D, Ferrannini E (2013). Human insulin resistance is associated with increased plasma levels of 12a-hydroxylated bile acids. Diabetes.

[54] Vincent RP, Omar S, Ghozlan S, Taylor DR, Cross G, Sherwood RA (2013). Higher circulating bile acid concentrations in obese patients with type 2 diabetes. Ann Clin Biochem.

[55] Pierre JF, Martinez KB, Ye H, Nadimpalli A, Morton TC, Yang J (2016). Activation of bile acid signaling improves metabolic phenotypes in high-fat diet-induced obese mice. Am J Physiol Gastrointest Liver Physiol.

[56] Feng Q, Liang S, Jia H, Stadlmayr A, Tang L, Lan Z (2015). Gut microbiome development along the colorectal adenoma-carcinoma sequence. Nat Commun.

[57] Labbé A, Ganopolsky JG, Martoni CJ, Prakash S, Jones ML (2014). Bacterial bile metabolising gene abundance in Crohn's, ulcerative colitis and type 2 diabetes metagenomes. PLoS One.

[58] Jie Z, Xia H, Zhong SL, Feng Q, Li S, Liang S (2017). The gut microbiome in atherosclerotic cardiovascular disease. Nat Commun.

[59] Fan Y, Li Y, Chen Y, Zhao YJ, Liu LW, Li J (2016). Comprehensive metabolomic characterization of coronary artery diseases. J Am Coll Cardiol.

[60] Charach G, Grosskopf I, Rabinovich A, Shochat M, Weintraub M, Rabinovich P (2011). The association of bile acid excretion and atherosclerotic coronary artery disease. Ther Adv Gastroenterol.

[61] Gylling H, Hallikainen M, Rajaratnam RA, Simonen P, Pihlajamaki J, Laakso M (2009). The metabolism of plant sterols is disturbed in postmenopausal women with coronary artery disease. Metab Clin Exp.

[62] Charach G, Rabinovich PD, Konikoff FM, Grosskopf I, Weintraub MS, Gilat T (1998). Decreased fecal bile acid output in patients with coronary atherosclerosis. J Med.

[63] Mayerhofer CCK, Ueland T, Broch K, Vincent RP, Cross GF, Dahl CP (2017). Increased secondary/primary bile acid ratio in chronic heart failure. J Card Fail.

[64] Camacho C, Coulouris G, Avagyan V, Ma N, Papadopoulos J, Bealer K (2009). BLAST+: architecture and applications. BMC Bioinf.

[65] PDBsum. http://www.ebi.ac.uk/thornton-srv/databases/pdbsum. Accessed 20 Oct 2018.

[66] Zhu W, Lomsadze A, Borodovsky M (2010). Ab initio gene identification in metagenomic sequences. Nucleic Acids Res.

[67] Rampelli S, Schnorr SL, Consolandi C, Turroni S, Severgnini M, Peano C (2015). Metagenome sequencing of the Hadza hunter-gatherer gut microbiota. Curr Biol.

[68] Qin N, Yang F, Li A, Prifti E, Chen Y, Shao L (2014). Alterations of the human gut microbiome in liver cirrhosis. Nature.

[69] Nishijima S, Suda W, Oshima K, Kim SW, Hirose Y, Morita H (2016). The gut microbiome of healthy Japanese and its microbial and functional uniqueness. DNA Res.

[70] Lim MY, Rho M, Song YM, Lee K, Sung J, Ko G. Stability of gut enterotypes in Korean monozygotic twins and their association with biomarkers and diet. Sci Rep. 2014;4:7348. 10.1038/srep07348 PMC4258686 25482875

[71] Le Chatelier E, Nielsen T, Qin J, Prifti E, Hildebrand F, Falony G (2013). Richness of human gut microbiome correlates with metabolic markers. Nature.

[72] Karlsson FH, Tremaroli V, Nookaew I, Bergstrom G, Behre CJ, Fagerberg B (2013). Gut metagenome in European women with normal, impaired and diabetic glucose control. Nature.

[73] Zeller G, Tap J, Voigt AY, Sunagawa S, Kultima JR, Costea PI (2014). Potential of fecal microbiota for early-stage detection of colorectal cancer. Mol Syst Biol.

[74] Obregon-Tito AJ, Tito RY, Metcalf J, Sankaranarayanan K, Clemente JC, Ursell LK (2015). Subsistence strategies in traditional societies distinguish gut microbiomes. Nat Commun.

[75] Martinez I, Stegen JC, Maldonado-Gomez MX, Eren AM, Siba PM, Greenhill AR (2015). The gut microbiota of rural Papua New Guineans: composition, diversity patterns, and ecological processes. Cell Rep.

[76] Lynch SV, Pedersen O (2016). The human intestinal microbiome in health and disease. N Engl J Med.

[77] Ruibang LBL, Yinlong X, Zhenyu L, et al. SOAPdenovo2: an empirically improved memory-efficient short-read de novo assembler. Gigascience. 2012;1:18. 10.1186/2047-217X-1-18 PMC3626529 23587118

[78] Kent WJ (2002). BLAT—the BLAST-like alignment tool. Genome Res.

[79] World Health Organization (WHO). http://www.who.int/en. Accessed 23 Mar 2017.

[80] Protein Homology/analogY Recognition Engine V 2.0. http://www.sbg.bio.ic.ac.uk/phyre2. Accessed 21 Oct 2018.

[81] Kelley LA, Mezulis S, Yates CM, Wass MN, Sternberg MJ (2015). The Phyre2 web portal for protein modeling, prediction and analysis. Nat Protoc.

[82] ZINC. http://zinc.docking.org. Accessed 21 Oct 2018.

[83] Morris GM, Goodsell DS, Halliday RS, Huey R, Hart WE, Belew RK (1998). Automated docking using a Lamarckian genetic algorithm and an empirical binding free energy function. J Comput Chem.

[84] Dong Z, Zhang J, Du G, Chen J, Li H, Lee B (2015). Periplasmic export of bile salt hydrolase in *Escherichia coli* by the twin-arginine signal peptides. Appl Biochem Biotechnol.

