# Supplementary material for: Taxonomic profiling and populational patterns of bacterial bile salt hydrolase (BSH) genes based on worldwide human gut microbiome
Source: Microbiome. 2019 Jan 23;7:9. doi: 10.1186/s40168-019-0628-3 (PMC6345003; doi:10.1186/s40168-019-0628-3)
Supplement: Supplementary file 1 — Figure S1. The enterohepatic circulation of bile acids. Figure S2. The analysis flowcharts. Figure S3. Genera characterization of BSHs obtained from NCBI database. Figure S4. The sequence identity of reported BSH sequences. Figure S5. Reported conserved residues of BSH in PDB database. Figure S6. Taxonomic identification of BSHs in HMP database. Figure S7. The Taxonomic identification and paralogs distribution of BSHs in HMP. Figure S8. Phylogenetic tree of BSHs in the gut microbiome of 11 populations. Figure S9. SDS-PAGE of BSH and Standard curve used in ninhydrin assay. Figure S10. Molecular docking results between BSH-T0 and bile acids. Figure S11. Molecular docking studies between BSH-T1 and bile acids. Figure S12. Molecular docking studies between BSH-T2 and bile acids. Figure S13. Molecular docking studies between BSH-T3 and bile acids. Figure S14. Molecular docking studies between BSH-T4 and bile acids. Figure S15. Molecular docking studies between BSH-T5 and bile acids. Figure S16. Molecular docking studies between BSH-T6 and bile acids. Figure S17. Molecular docking studies between BSH-T7 and bile acids. Figure S18. Enzyme activity comparisons between substrates in each BSH phylotype. Figure S19. Relationship between average RA of BSHs with WHO released phenotypes in 11 populations. Figure S20. The relative abundance of BSHs among case-control cohorts. Figure S21. Standard curve of bile acids used in LC-MS/MS. Figure S22. Typical LC spectrum of different bile acids. (PDF 4413 kb) [file 40168_2019_628_MOESM1_ESM.pdf]

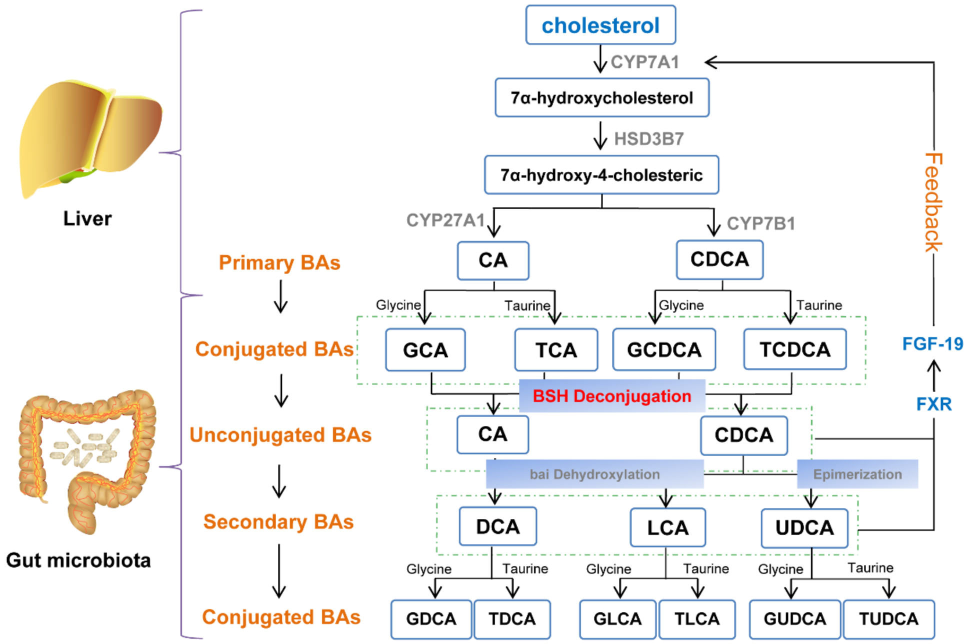

Figure S1 The enterohepatic circulation of bile acids. In the liver, cholesterol is converted to 7α-hydroxycholesterol by CYP7A1. The 3β-hydroxysteroid dehydrogenase (HSD3B7) converts 7α-hydroxycholesterol to 7α-hydroxy-4-cholesteric, which eventually is converted to primary bile acids (BAs) by CYP7A1 and CYP27B1. The primary BAs (CA and CDCA) usually conjugated with the glycine or taurine to conjugated BAs (GCA, TCA, GCDCA and TCDCA), and transfer to intestine. BSH in gut microbiota would catalyze the hydrolysis of conjugated BAs into deconjugated BAs. Then, microbial bai dehydroxylase removes a hydroxyl group from C-7 and converts CA to DCA or CDCA to LCA and UDCA. These secondary BAs could active the feedback pathway to preserve the balance of metabolism of BAs by FXR . Meanwhile, the DCA, LCA and UDCA could conjugate with glycine and taurine to conjugated BAs (GDCA, TDCA, GLCA, TLCA, GUDCA and TUDCA). CA, Cholic acid; CDCA, Chenodeoxycholic acid; GCA, Glycocholic acid; TCA, Taurocholic acid; GCDCA, Glycochenodeoxycholic acid; TCDCA, Taurochnodeoxycholic acid; DCA, Deoxycholic acid; LCA, Lithocholic acid; UDCA, Ursodeoxycholic acid; GDCA, Glycine deoxycholic acid; TDCA, Taurodeoxycholic acid; GLDCA, Glycolithocholic acid; TLDCA, Taurolithocholic acid; GUDCA, Glycoursodeoxycholicacid;TUDCA, tauroursodeoxycholic acid.

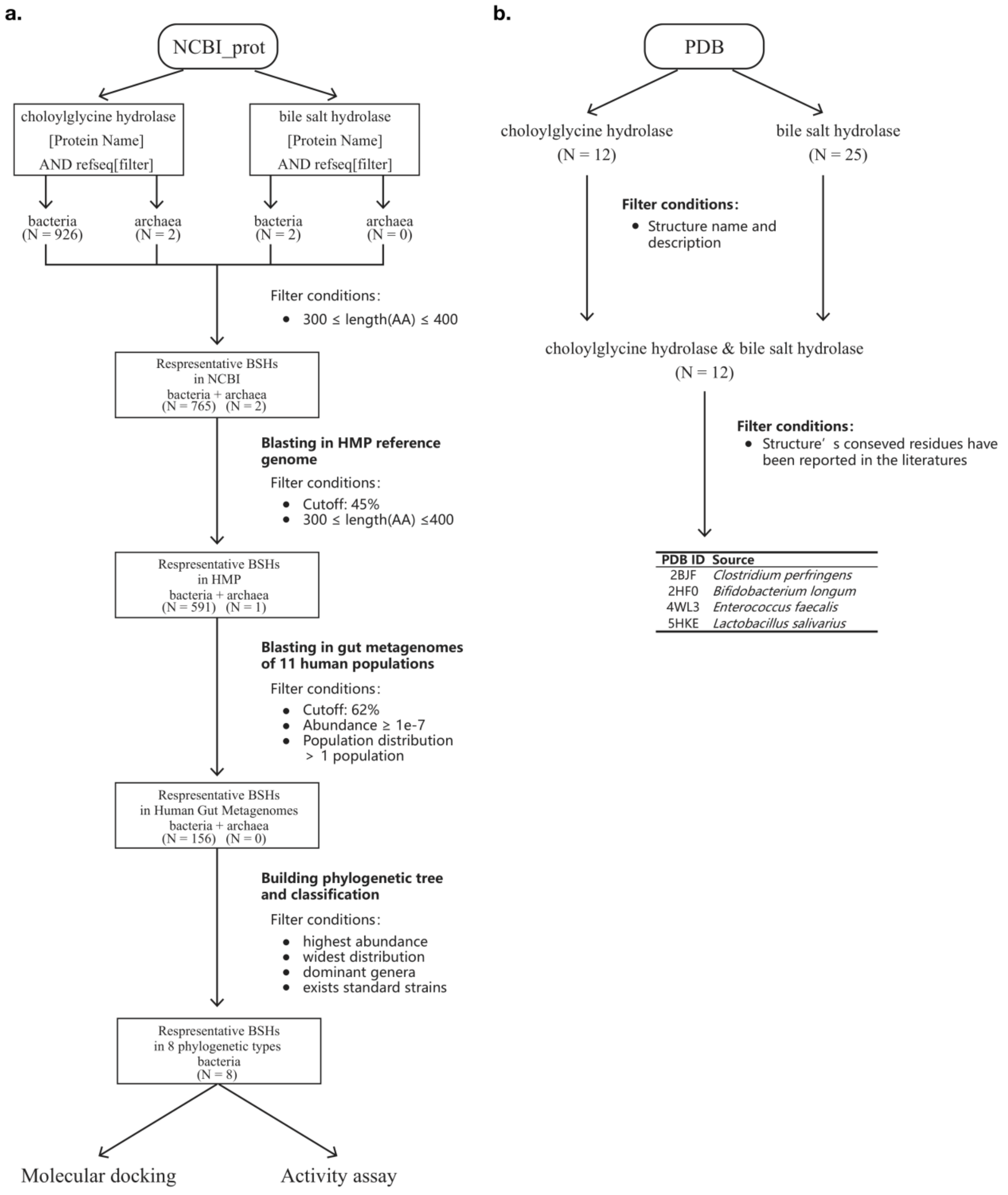

Figure S2 The analysis flowcharts. a The screening process of BSH sequence for taxonomic and activity identification. **b** The filter procedures of typical BSH structures.

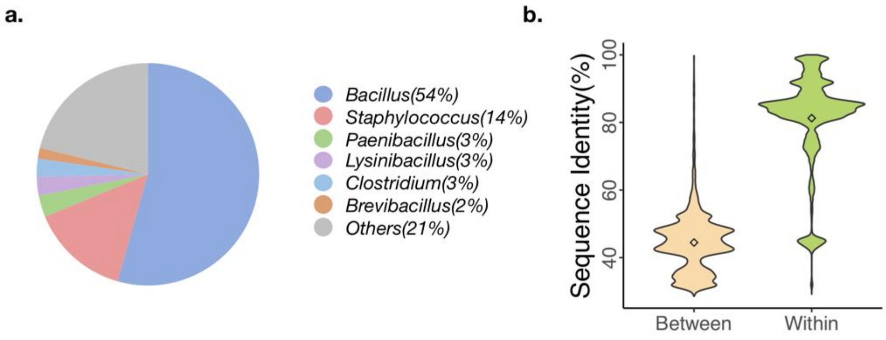

Figure S3 Genera characterization of BSHs obtained from NCBI database. a The proportion of 765 reported BSHs containing genus. **b** The identity of 765 reported BSHs between genus and within genus.

a.

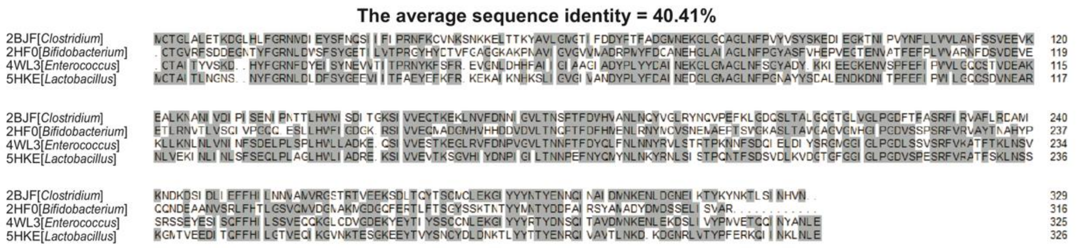

b.

PDB ID	Sequence alignment, identity(%)		
	2BJF	2HF0	4WL3
2HF0	35.76		
4WL3	43.46	36.74	
5HKE	37.27	34.91	54.29

Figure S4 The sequence identity of reported BSH sequences. a Multiple sequences alignment of reported BSHs. Grey backgrounds indicate an average identity ≥50% among three BSHs. **b** The pairwise sequence identity of four reported BSH sequences.

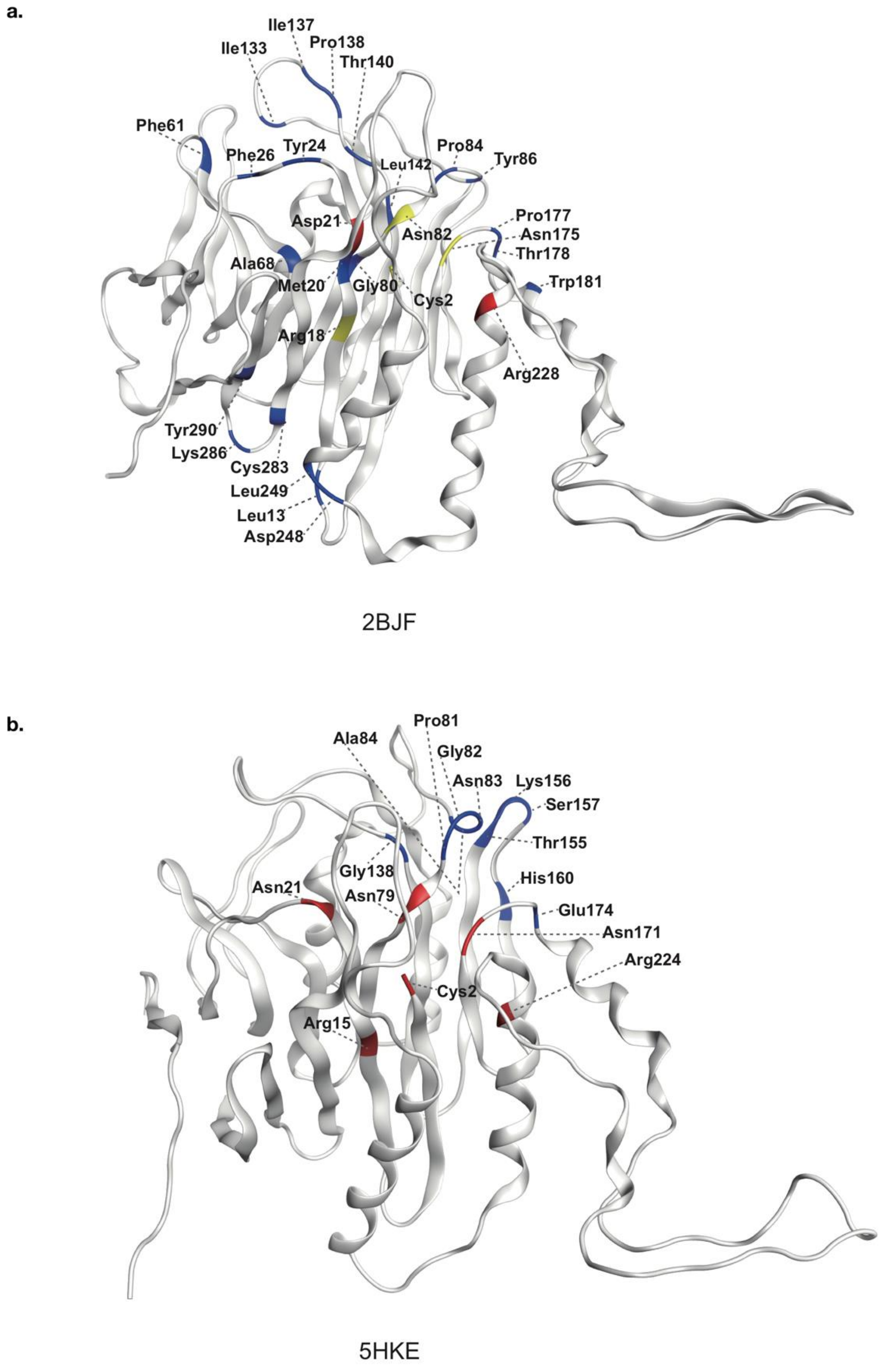

Figure S5 Reported conserved residues of BSH in PDB database. a 3D structure of CpBSH (PDB ID 2BJF) and LsBSH (PDB ID 5HKE) with their residues reported in the literature (red), in the PDBsum (blue) and both in the literature and PDBsum (yellow).

	Phylum	Order	Family	Genus	No.
Bacteria	Actinobacteria	Bifidobacteriales	Bifidobacteriaceae	Bifidobacterium	17
		Corynebacteriales	Gordoniaceae	Gordonia	1
			Mycobacteriaceae	Mycobacterium	5
		Micrococcales	Micrococcaceae	Paenarthrobacter	1
		Nakamurellales	Nakamurellaceae	Nakamurella	1
		Pseudonocardiales	Pseudonocardiaceae	Saccharopolyspora	1
		Streptomycetales	Streptomycetaceae	Streptomyces	1
		Coriobacteriales	Coriobacteriaceae	Collinsella	1
		Eggerthellales	Eggerthellaceae	Slackia	1
	Bacteroidetes	Bacteroidales	Bacteroidaceae	Bacteroides	78
			Porphyromonadaceae	Parabacteroides	10
			Rikenellaceae	Alistipes	1
		Cytophagales	Cytophagaceae	Cytophaga	1
				Microscilla	1
		Flavobacteriales	Flavobacteriaceae	Chryseobacterium	1
				Flavobacterium	1
		Sphingobacteriales	Sphingobacteriaceae	Sphingobacterium	2
	Chlamydiae	Chlamydiales	Waddliaceae	Waddlia	1
	Cyanobacteria	Gloeobacterales		Gloeobacter	1
		Chroococcales	Unassigned	Cyanothece	1
				Synechococcus	2
				Synechocystis	1
	Euryarchaeota	Methanobacteriales	Methanobacteriaceae	Methanosphaera	1
	Firmicutes	Bacillales	Bacillaceae	Bacillus	120
				Oceanobacillus	2
			Listeriaceae	Listeria	11
			Staphylococcaceae	Macrococcus	1
				Staphylococcus	49
			Unassigned	Exiguobacterium	2
		Lactobacillales	Enterococcaceae	Enterococcus	40
			Lactobacillaceae	Lactobacillus	39
			Streptococcaceae	Streptococcus	4
		Clostridiales	Clostridiaceae	Clostridium	23
				Hungatella	1
			Eubacteriaceae	Anaerofustis	1
				Eubacterium	6
			Lachnospiraceae	Anaerostipes	1
				Blautia	5
				Butyrivibrio	2
				Cellulosilyticum	1
				Coprococcus	2
				Dorea	1
				Lachnoclostridium	4
				Marvinbryantia	2
				Oribacterium	1
				Roseburia	4
				Tyzzerella	2
				Unassigned	1
			Peptococcaceae	Desulfitobacterium	1
			Peptostreptococcaceae	Intestinibacter	1
				Peptoclostridium	12
			Ruminococcaceae	Anaerotruncus	1
				Faecalibacterium	1
				Ruminiclostridium	1
				Ruminococcus	1
			Unassigned	Unassigned	3
		Erysipelotrichales	Erysipelotrichaceae	Catenibacterium	1
				Coprobacillus	1
				Erysipelatoclostridium	1
				Holdemanella	1
				Turicibacter	1
				Unassigned	1
		Tissierellales	Peptoniphilaceae	Peptoniphilus	1
	Fusobacteria	Fusobacteriales	Fusobacteriaceae	Fusobacterium	5
	Planctomycetes	Planctomycetales	Planctomycetaceae	Blastopirellula	2
				Gemmata	1
				Gimesia	1
				Pirellula	1
				Planctopirus	1
				Rhodopirellula	1
	Proteobacteria	Acidithiobacillales	Acidithiobacillaceae	Acidithiobacillus	1
		Rhizobiales	Beijerinckiaceae	Beijerinckia	1
			Bradyrhizobiaceae	Nitrobacter	2
				Oligotropha	1
				Rhodopseudomonas	5
			Hyphomicrobiaceae	Hyphomicrobium	1
				Rhodomicrobium	1
			Methylobacteriaceae	Methylobacterium	3
			Phyllobacteriaceae	Mesorhizobium	1
			Rhizobiaceae	Agrobacterium	1
				Rhizobium	1
			Xanthobacteraceae	Azorhizobium	1
				Starkeya	1
				Xanthobacter	1
		Rhodobacterales	Rhodobacteraceae	Labrenzia	1
				Roseobacter	1
		Rhodospirillales	Acetobacteraceae	Gluconacetobacter	1
		Rickettsiales	Rickettsiaceae	Rickettsia	1
		Burkholderiales	Alcaligenaceae	Bordetella	4
			Burkholderiaceae	Burkholderia	3
				Polynucleobacter	1
			Comamonadaceae	Comamonas	2
				Delftia	1
			Oxalobacteraceae	Oxalobacter	2
		Bdellovibrionales	Bdellovibrionaceae	Bdellovibrio	1
		Desulfobacterales	Desulfobulbaceae	Desulfotalea	2
		Desulfovibrionales	Desulfovibrionaceae	Desulfovibrio	1
		Aeromonadales	Aeromonadaceae	Aeromonas	1
		Alteromonadales	Shewanellaceae	Shewanella	2
		Chromatiales	Halothiobacillaceae	Halothiobacillus	1
		Enterobacteriales	Enterobacteriaceae	Escherichia	1
				Pectobacterium	3
				Proteus	2
				Providencia	4
				Yersinia	5
		Legionellales	Legionellaceae	Legionella	1
		Pseudomonadales	Moraxellaceae	Acinetobacter	4
			Pseudomonadaceae	Pseudomonas	2
		Vibrionales	Vibrionaceae	Aliivibrio	1
				Photobacterium	3
				Vibrio	14
		Xanthomonadales	Xanthomonadaceae	Stenotrophomonas	2
	Spirochaetes	Brachyspirales	Brachyspiraceae	Brachyspira	3
		Spirochaetales	Spirochaetaceae	Treponema	1
	Synergistetes	Synergistales	Synergistaceae	Dethiosulfovibrio	1
				Pyramidobacter	1
	Verrucomicrobia	Verrucomicrobiales	Verrucomicrobiaceae	Verrucomicrobium	1
Archaea	Euryarchaeota	Natrialbales	Natrialbaceae	Haloterrigena	1

Figure S6 Taxonomic identification of BSHs in HMP database. The numbers in last column indicate the number of BSHs in each genus.

Phylum	Class	Order	Family	Genus	Number of paralogs		
					NP=2	NP=3	NP=4
Actinobacteria	*Actinobacteria*	*Bifidobacteriales*	*Bifidobacteriaceae*	*Bifidobacterium*	1	-	-
Bacteroidetes	*Bacteroidia*	*Bacteroidales*	*Bacteroidaceae*	*Bacteroides*	15	9	3
			Porphyromonadaceae	*Parabacteroides*	4	-	-
Firmicutes	*Bacilli*	*Bacillales*	*Bacillaceae*	*Bacillus*	41	5	-
				Oceanobacillus	1	-	-
			Staphylococcaceae	*Staphylococcus*	6	-	-
		Lactobacillales	*Enterococcaceae*	*Enterococcus*	13	-	-
			Lactobacillaceae	*Lactobacillus*	6	-	-
	Clostridia	*Clostridiales*	*Clostridiaceae*	*Clostridium*	5	-	-
			Eubacteriaceae	*Eubacterium*	3	-	-
			Lachnospiraceae	*Blautia*	2	-	-
				Butyrivibrio	1	-	-
				Lachnoclostridium	1	-	-
				Marvinbryantia	1	-	-
				Roseburia	-	1	-
				Tyzzerella	1	-	-
			Unclassified Clostridiales	*Unclassified Clostridiales*	1	-	-
Planctomycetes	*Planctomycetia*	*Planctomycetales*	*Planctomycetaceae*	*Blastopirellula*	1	-	-
Proteobacteria	*Gammaproteobacteria*	*Vibrionales*	*Vibrionaceae*	*Photobacterium*	1	-	-
	Deltaproteobacteria	*Desulfobacterales*	*Desulfobulbaceae*	*Desulfotalea*	1	-	-

Figure S7 The Taxonomic identification and paralogs distribution of BSHs in HMP. The numbers in last three columns indicate the number of strains containing BSH paralogs.

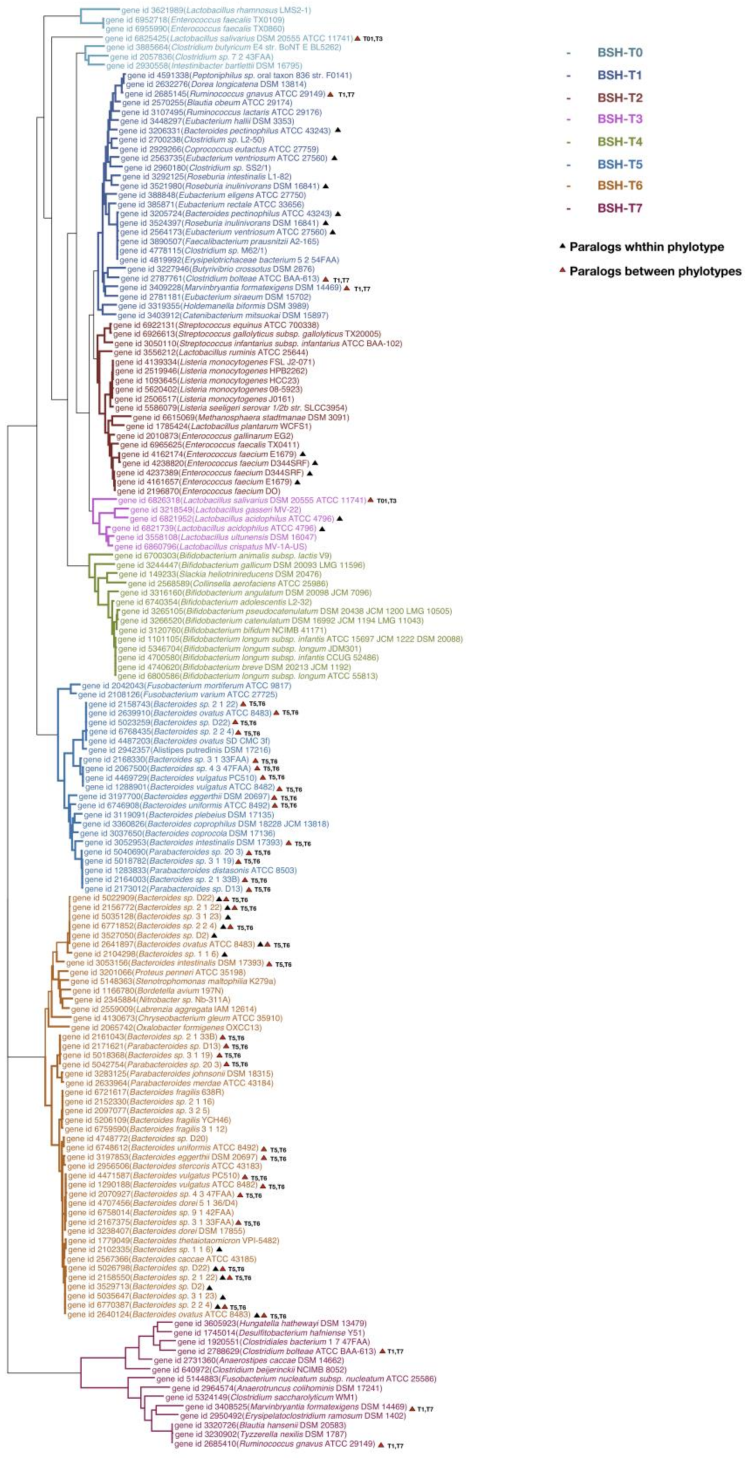

Figure S8 Phylogenetic tree of BSHs in the gut micorbiome of 11 populations. The id and strain name of BSHs are provided. Different color represents different phylotype of reclassify BSHs, from top to bottom are BSH-T0, BSH-T1, BSH-T2, BSH-T3, BSH-T4, BSH-T5, BSH-T6 and BSH- T7, respectively. Triangles indicate the strains with paralogs.

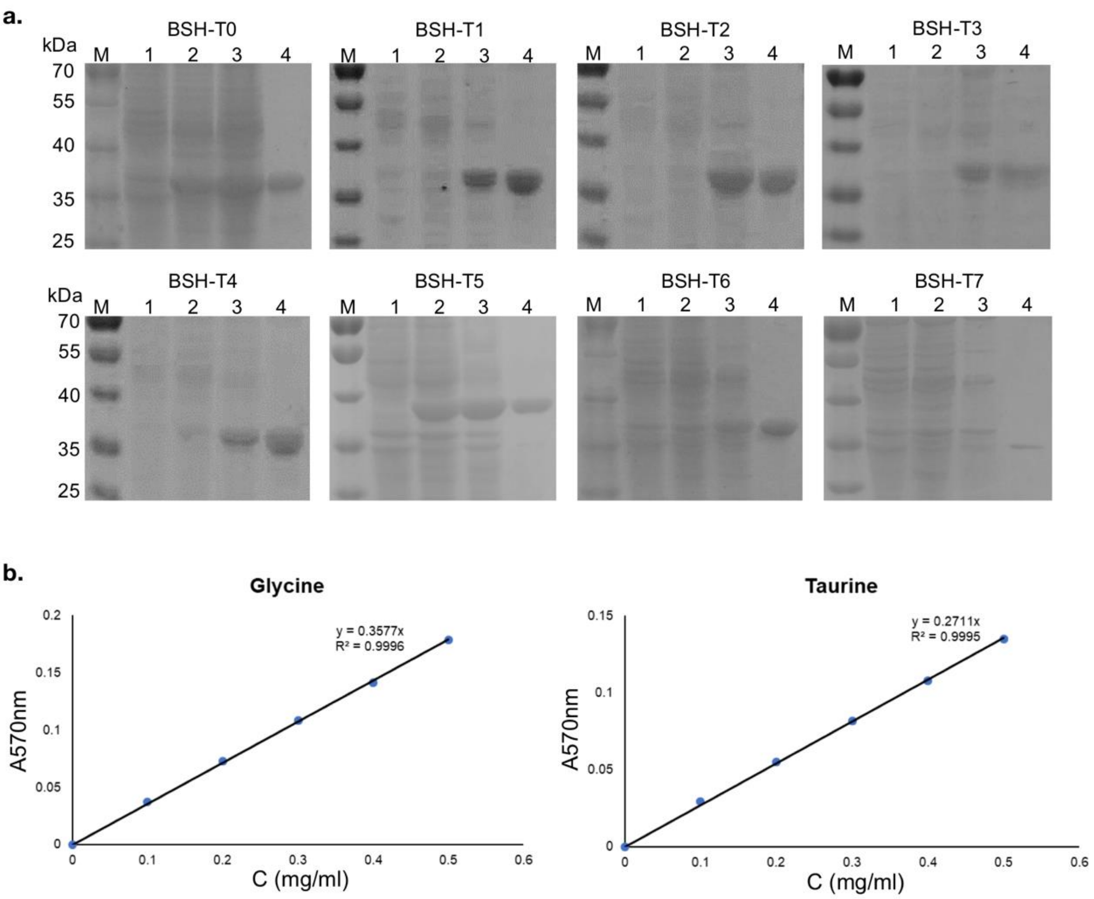

Figure S9 SDS-PAGE of BSH and Standard curve used in ninhydrin assay. a SDS-PAGE of BSH protein. M: Marker; Lane 1: *E.coli* BL21(DE3); Lane 2: *E.coli* BL21(DE3)-*bsh* (-IPTG), Lane 3: *E.coli* BL21(DE3)-*bsh* (+IPTG), Lane 4: Purified BSH. **b** Standard curve of glycine and taurine.

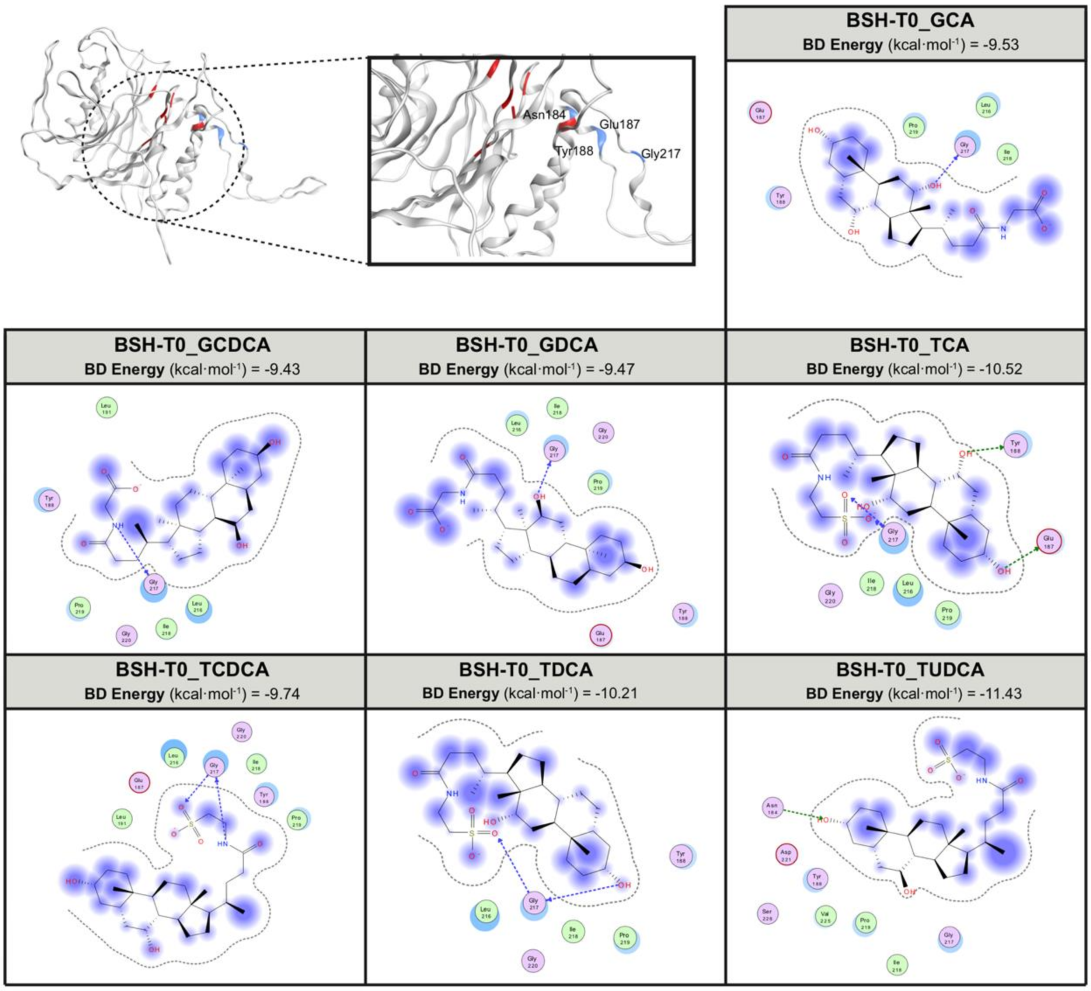

	GCA	GCDCA	GDCA	TCA	TCDCA	TDCA	TUDCA
**Residue contacts : to ligand**	Gly217	Gly217	Gly217	Glu187<br>Tyr188<br>Gly217	Gly217	Gly217	Asn184

Figure S10 Molecular docking results between BSH-T0 and bile acids. The predicted binding models of BSH-T0 with GCA, GCDCA, GDCA, TCA, TCDCA, TDCA and TUDCA. The residues contact to ligand showed in the below table.

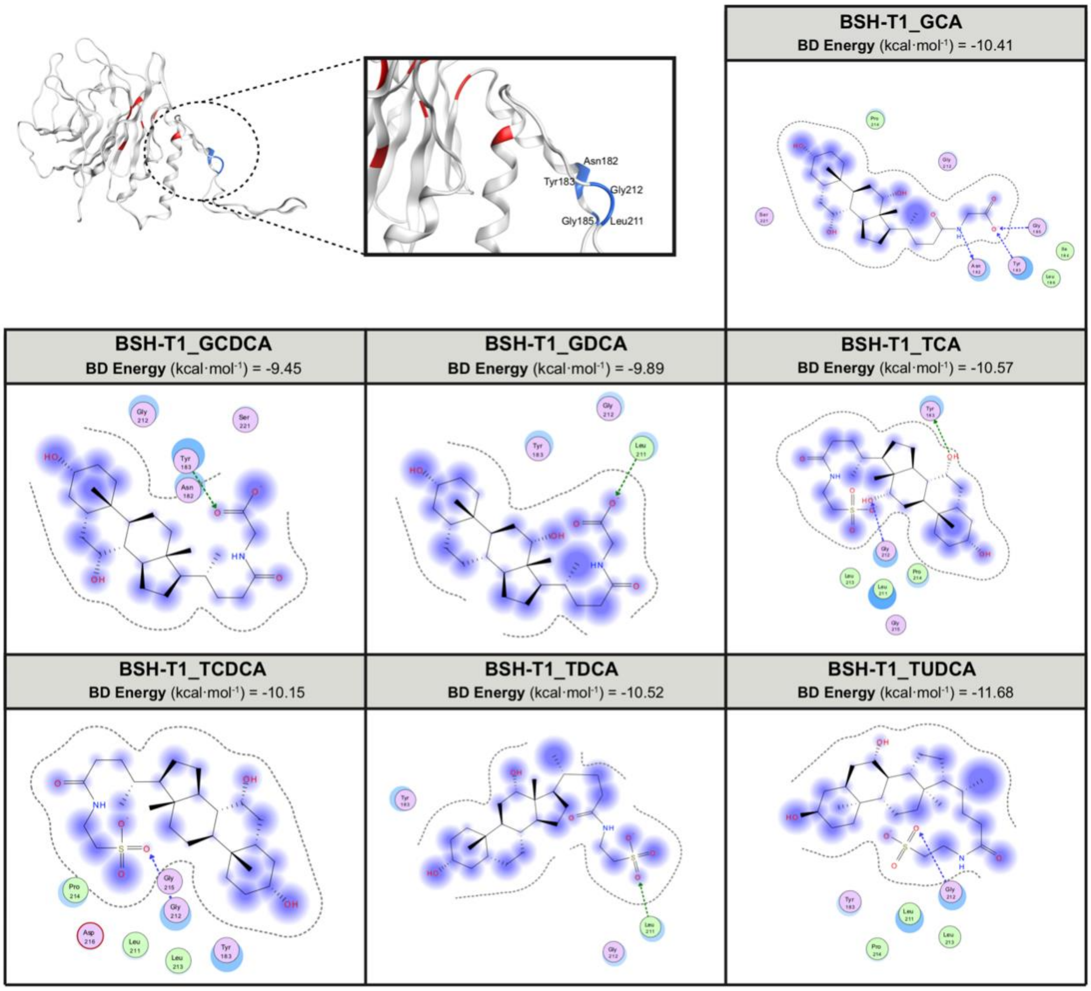

	GCA	GCDCA	GDCA	TCA	TCDCA	TDCA	TUDCA
**Residue contacts : to ligand**	Asn182 Tyr183 Gly185	Tyr183	Leu211	Tyr183 Gly212	Gly212	Leu211	Gly212

Figure S11 Molecular docking studies between BSH-T1 and bile acids. The predicted binding models of BSH-T1 with GCA, GCDCA, GDCA, TCA, TCDCA, TDCA and TUDCA. The residues contact to ligand showed in the below table.

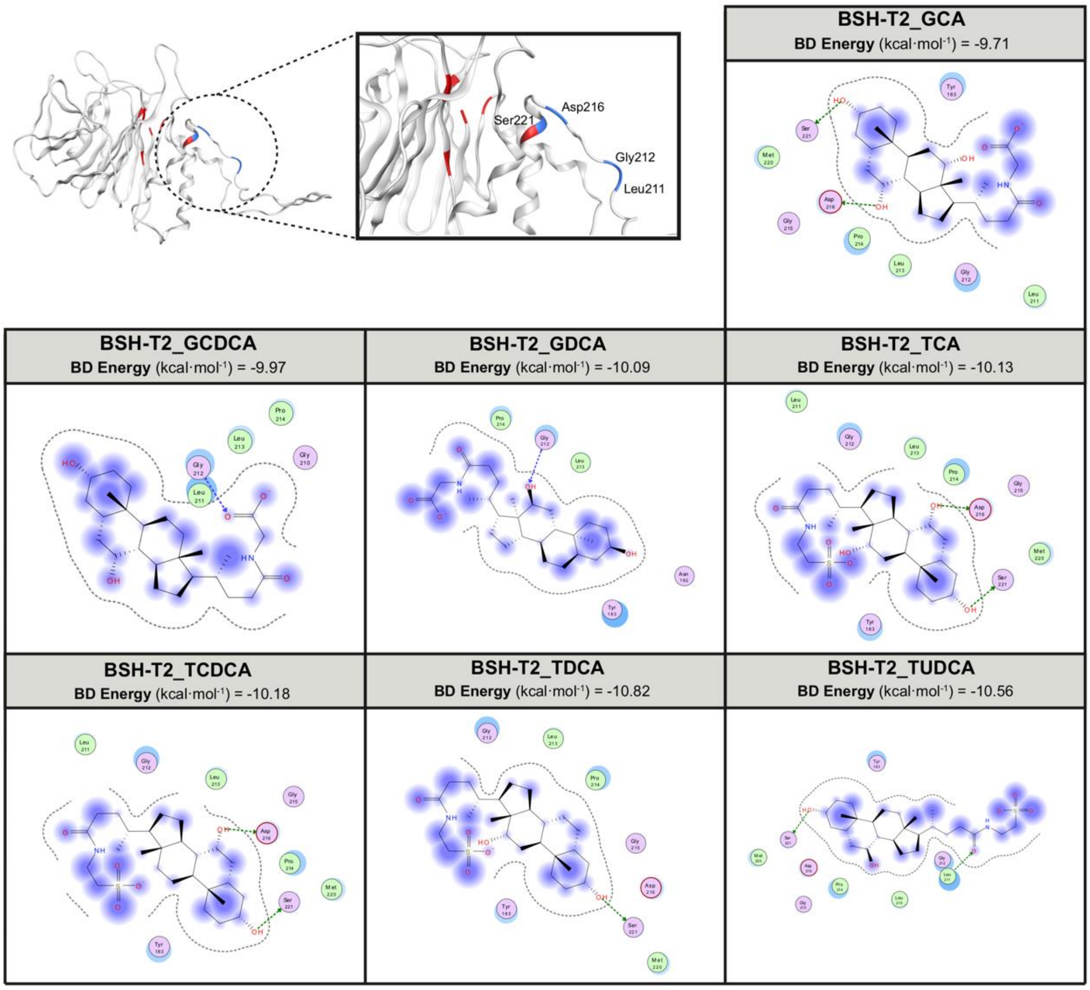

	GCA	GCDCA	GDCA	TCA	TCDCA	TDCA	TUDCA
**Residue contacts : to ligand**	Asp216 Ser221	Gly212	Gly212	Asp216 Ser221	Asp216 Ser221	Ser221	Leu211 Ser221

Figure S12 Molecular docking studies between BSH-T2 and bile acids. The predicted binding models of BSH-T2 with GCA, GCDCA, GDCA, TCA, TCDCA, TDCA and TUDCA. The residues contact to ligand showed in the below table.

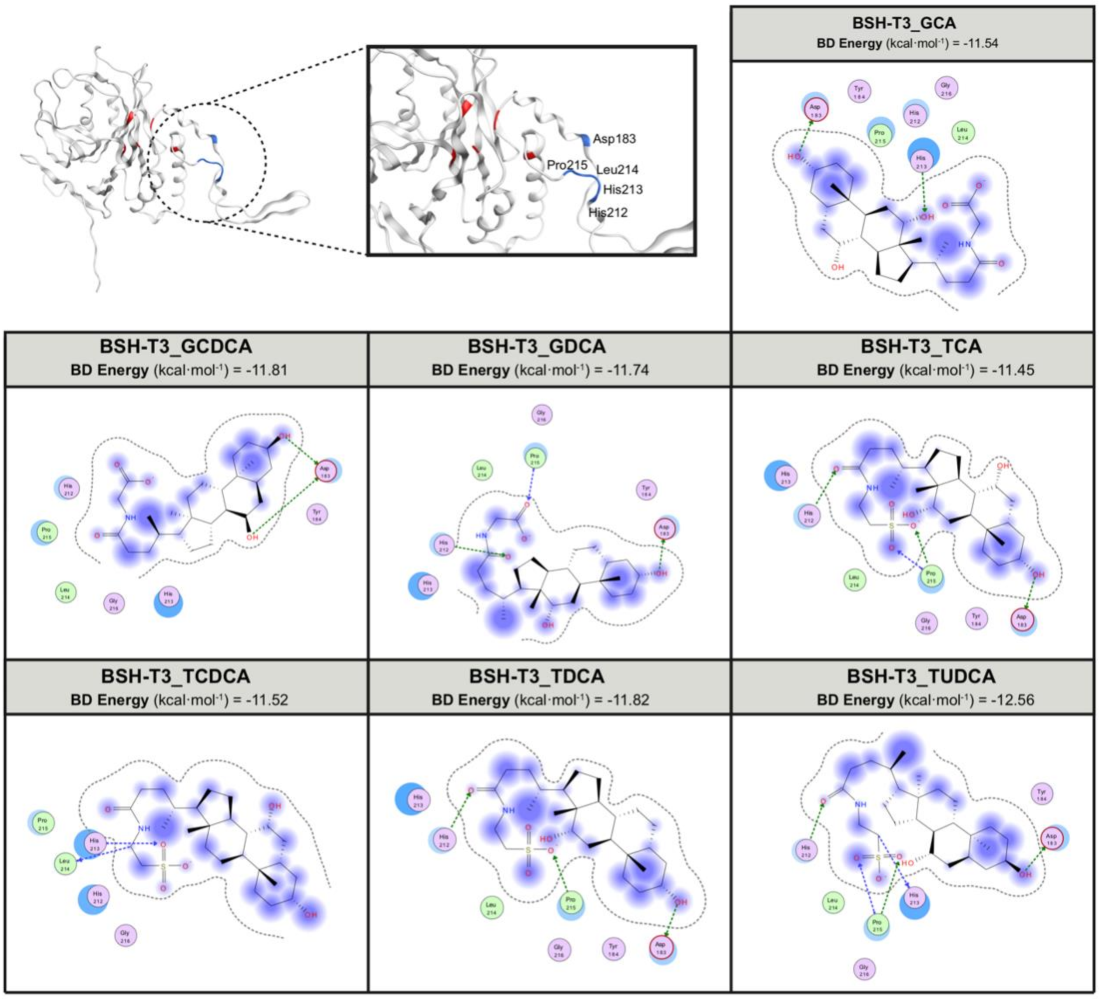

	GCA	GCDCA	GDCA	TCA	TCDCA	TDCA	TUDCA
**Residue contacts : to ligand**	Asp183 His213	Asp183	Asp183 His212 Pro215	Asp183 His212 Pro215	His213 Leu214	Asp183 His212 Pro215	Asp183 His213 Pro215

Figure S13 Molecular docking studies between BSH-T3 and bile acids. The predicted binding models of BSH-T3 with GCA, GCDCA, GDCA, TCA, TCDCA, TDCA and TUDCA. The residues contact to ligand showed in the below table.

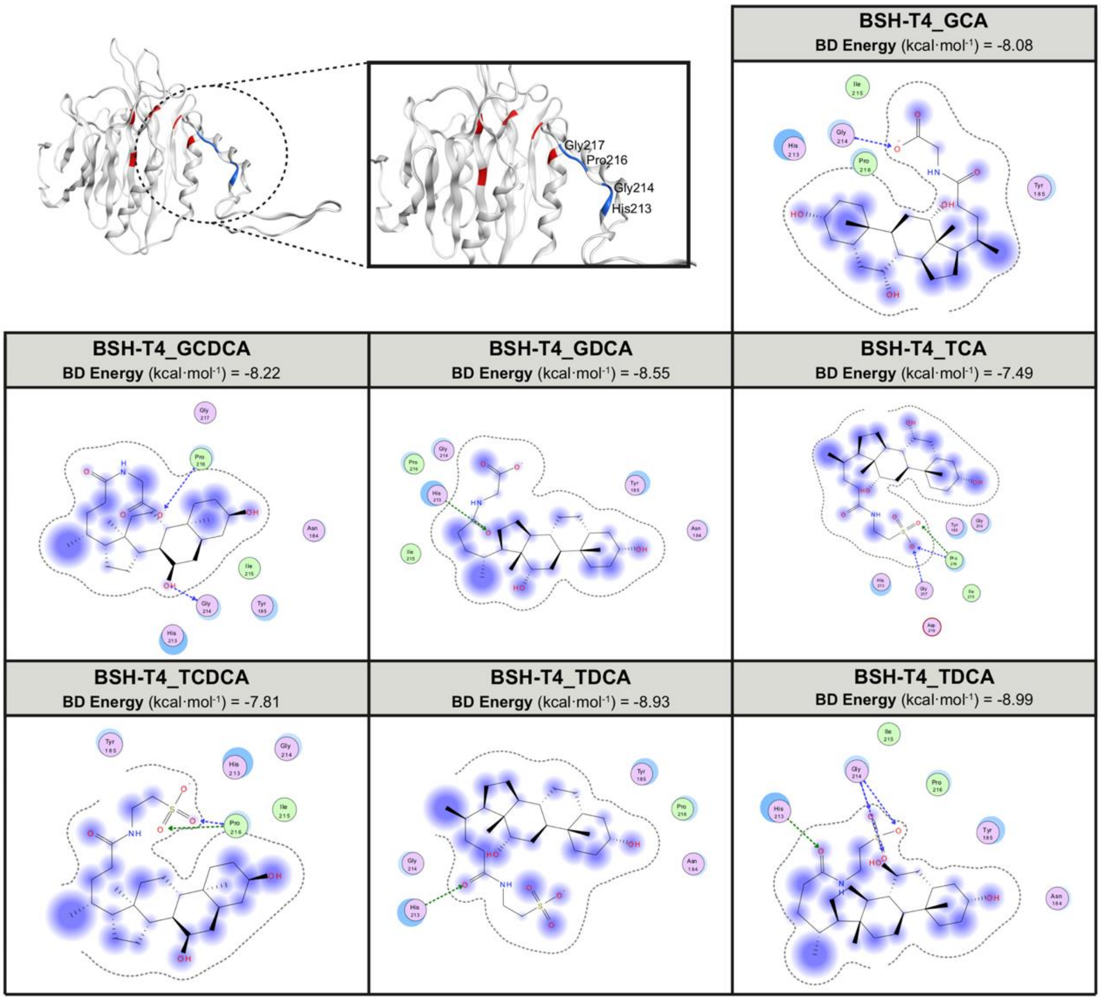

	GCA	GCDCA	GDCA	TCA	TCDCA	TDCA	TUDCA
**Residue contacts : to ligand**	Gly214	Gly214<br>Pro216	His213	Pro216<br>Gly217	Pro216	His213	His213<br>Gly214

Figure S14 Molecular docking studies between BSH-T4 and bile acids. The predicted binding models of BSH-T4 with GCA, GCDCA, GDCA, TCA, TCDCA, TDCA and TUDCA. The residues contact to ligand showed in the below table.

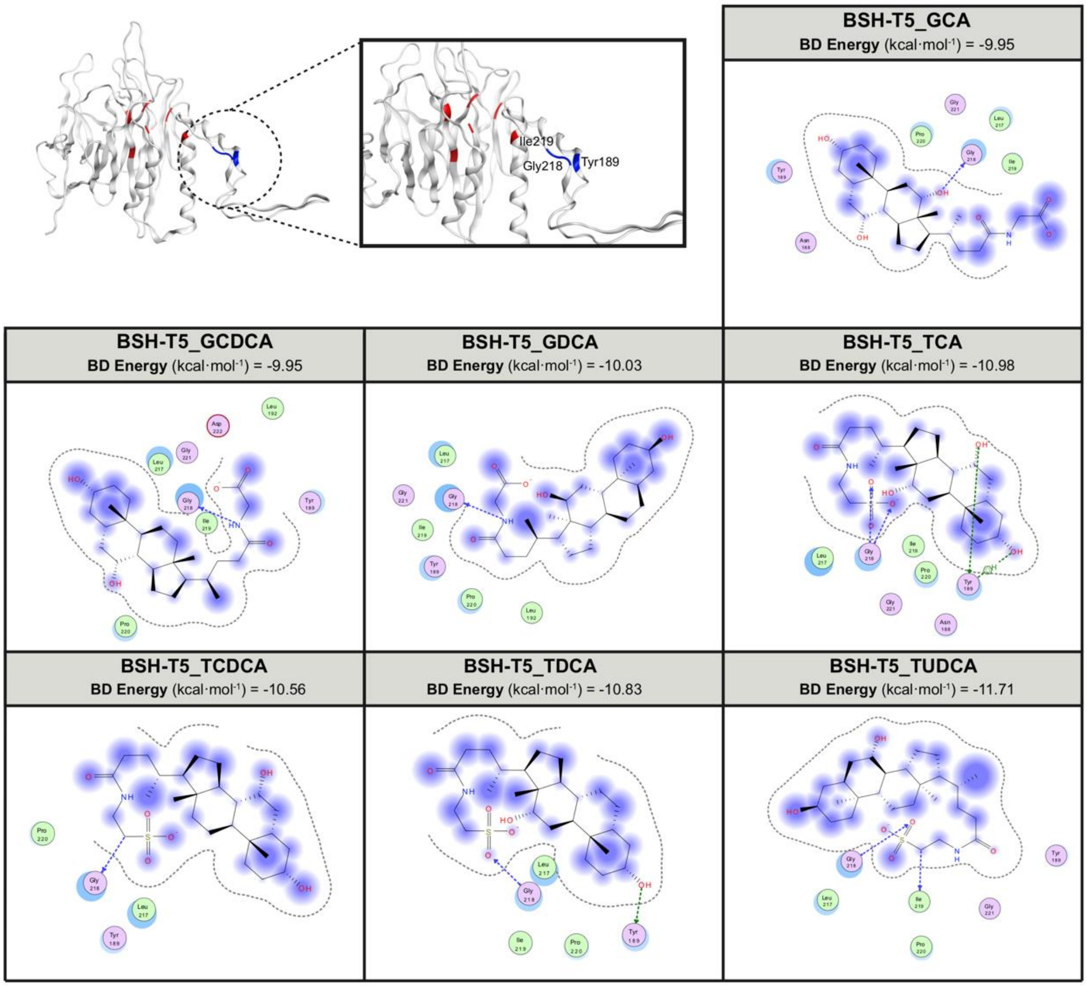

	GCA	GCDCA	GDCA	TCA	TCDCA	TDCA	TUDCA
**Residue contacts : to ligand**	Gly218	Gly218	Gly218	Tyr189<br>Gly218	Gly218	Tyr189<br>Gly218	Gly218<br>Ile219

Figure S15 Molecular docking studies between BSH-T5 and bile acids. The predicted binding models of BSH-T5 with GCA, GCDCA, GDCA, TCA, TCDCA, TDCA and TUDCA. The residues contact to ligand showed in the below table.

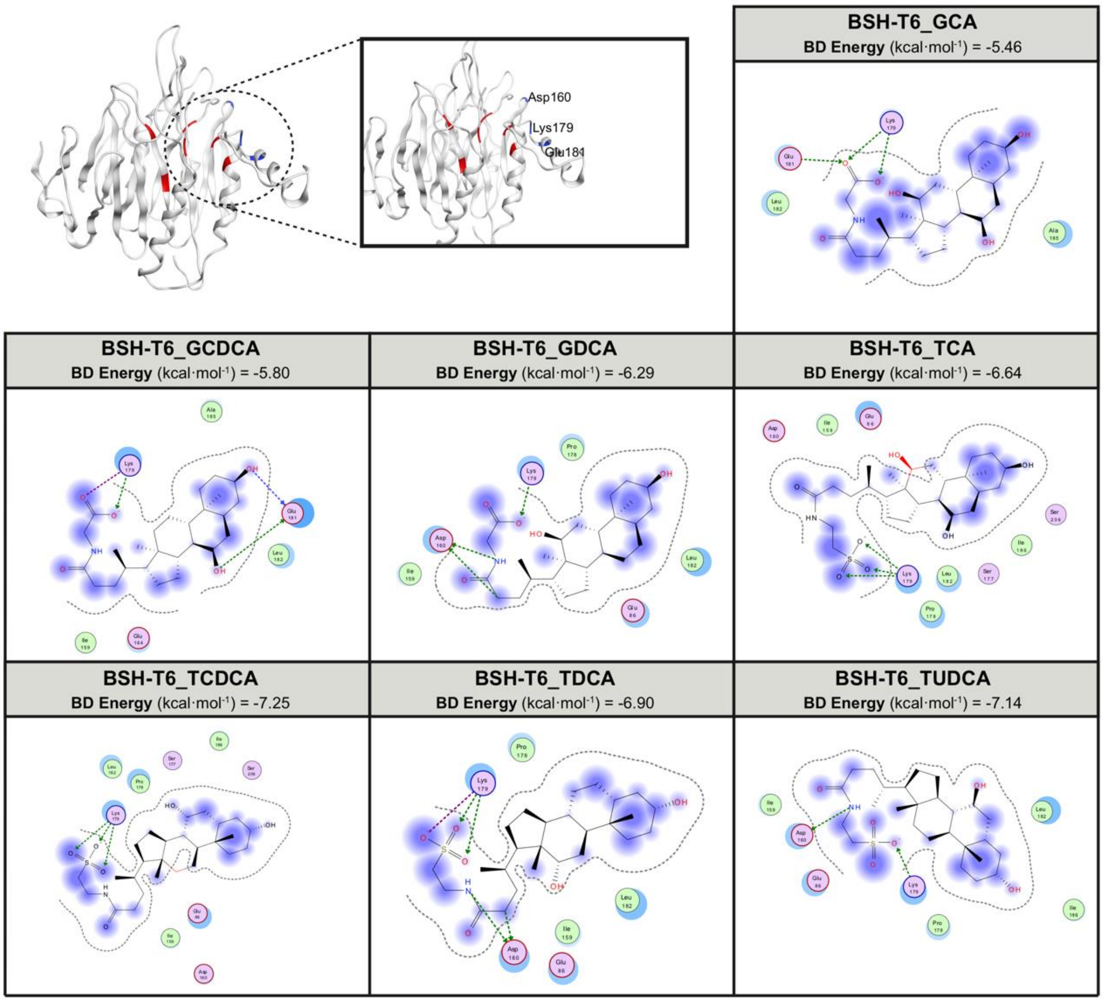

	GCA	GCDCA	GDCA	TCA	TCDCA	TDCA	TUDCA
**Residue contacts : to ligand**	Lys179<br>Glu181	Lys179<br>Glu181	Asp160<br>Lys179	Lys179	Lys179	Asp160<br>Lys179	Asp160<br>Lys179

Figure S16 Molecular docking studies between BSH-T6 and bile acids. The predicted binding models of BSH-T6 with GCA, GCDCA, GDCA, TCA, TCDCA, TDCA and TUDCA. The residues contact to ligand showed in the below table.

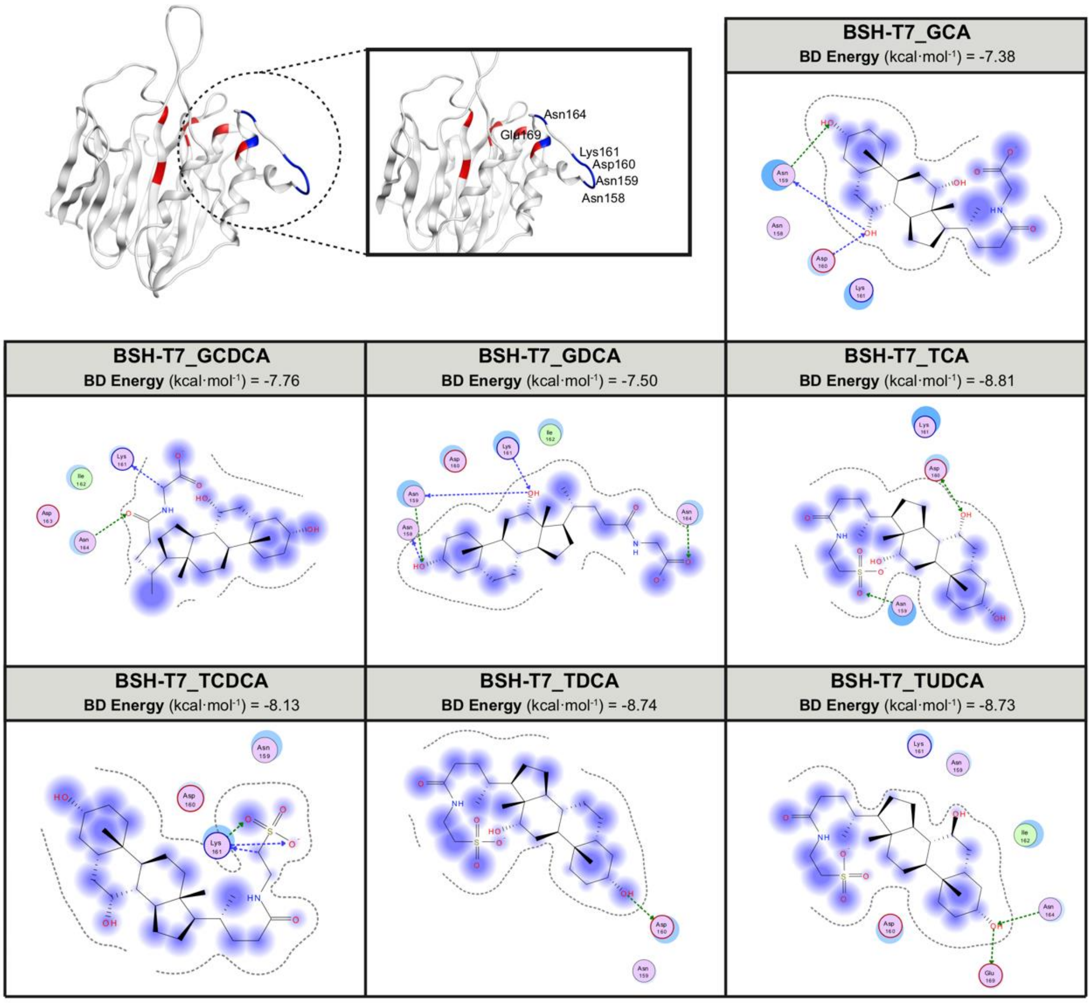

	GCA	GCDCA	GDCA	TCA	TCDCA	TDCA	TUDCA
**Residue contacts : to ligand**	Asn159<br>Asp160	Lys161<br>Asn164	Asn158<br>Asn159<br>Lys161<br>Asn164	Asn159<br>Asp160	Lys161	Asp160	Asn164<br>Glu169

Figure S17 Molecular docking studies between BSH-T7 and bile acids. The predicted binding models of BSH-T7 with GCA, GCDCA, GDCA, TCA, TCDCA, TDCA and TUDCA. The residues contact to ligand showed in the below table.

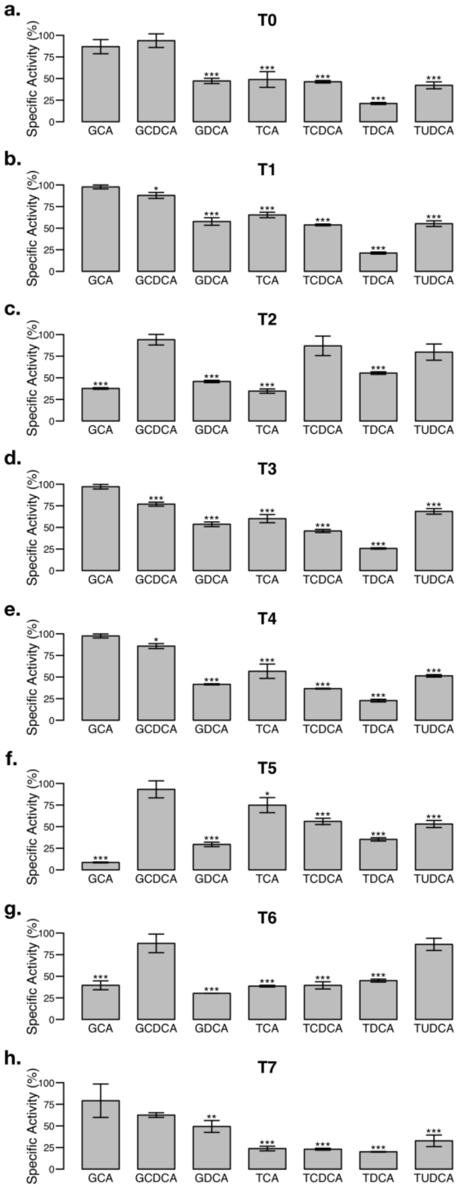

T0

	GCDCA	GDCA	TCA	TCDCA	TDCA	TUDCA
GCA	–	***	***	***	***	***
GCDCA		***	***	***	***	***
GDCA			–	–	**	–
TCA				–	***	–
TCDCA					**	–
TDCA						**

T1

	GCDCA	GDCA	TCA	TCDCA	TDCA	TUDCA
GCA	*	***	***	***	***	***
GCDCA		***	***	***	***	***
GDCA			–	–	***	–
TCA				**	***	*
TCDCA					***	–
TDCA						***

T2

	GCDCA	GDCA	TCA	TCDCA	TDCA	TUDCA
GCA	***	–	–	***	*	***
GCDCA		***	***	–	***	–
GDCA			–	***	–	***
TCA				***	*	***
TCDCA					***	–
TDCA						**

T3

	GCDCA	GDCA	TCA	TCDCA	TDCA	TUDCA
GCA	***	***	***	***	***	***
GCDCA		***	***	***	***	*
GDCA			–	–	***	***
TCA				***	***	*
TCDCA					***	***
TDCA						***

T4

	GCDCA	GDCA	TCA	TCDCA	TDCA	TUDCA
GCA	*	***	***	***	***	***
GCDCA		***	***	***	***	***
GDCA			**	–	***	–
TCA				***	***	–
TCDCA					**	**
TDCA						***

T5

	GCDCA	GDCA	TCA	TCDCA	TDCA	TUDCA
GCA	***	**	***	***	***	***
GCDCA		***	*	***	***	***
GDCA			***	***	–	**
TCA				*	***	**
TCDCA					**	–
TDCA						*

T6

	GCDCA	GDCA	TCA	TCDCA	TDCA	TUDCA
GCA	***	–	–	–	–	***
GCDCA		***	***	***	***	–
GDCA			–	–	–	***
TCA				–	–	***
TCDCA					–	***
TDCA						***

T7

	GCDCA	GDCA	TCA	TCDCA	TDCA	TUDCA
GCA	–	**	***	***	***	***
GCDCA		–	***	***	***	**
GDCA			*	*	**	–
TCA				–	–	–
TCDCA					–	–
TDCA						–

Figure S18 Enzyme activity comparisons between substrates in each BSH phylotype. The specific activity of an enzyme per milligram of total protein, using the highest enzyme activity as 100% for each BSH phylotype. **a** BSH-T0; **b** BSH-T1; **c** BSH-T2; **d** BSH-T3; **e** BSH-T4; **f** BSH-T5; **g** BSH-T6 and **h** BSH-T7. p value was analyzed by single-factor ANOVA with Tukey HSD test, * $p < 0.05$, ** $p < 0.01$, *** $p < 0.001$, versus the substrate which shown highest enzyme activity in each BSH. Details shown in Table S8. GCA, Glycocholic acid; GCDCA, Glycochenodeoxycholic acid; GDCA, Glycine deoxycholic acid; TCA, Taurocholic acid; TCDCA, Taurochnodeoxycholic acid; TDCA, Taurodeoxycholic acid; TUDCA, Tauroursodeoxycholic acid.

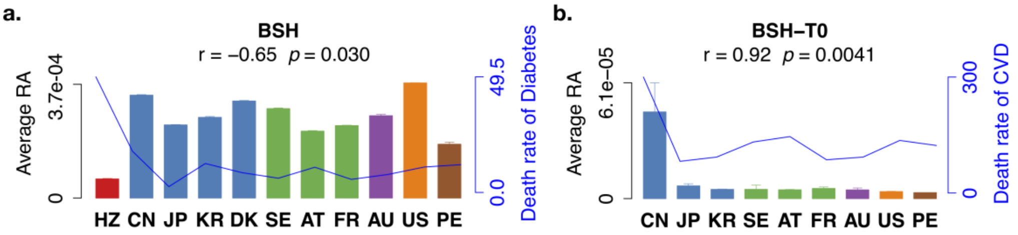

c.

	BSH	Population	Correlation			
			Death rate of diabetes	Death rate of CVD	Mean blood cholesterol	BMI of obesity
1	All	HZ、CN、JP、KR DK、SE、AT、FR AU、US、PE	**r = −0.65 *p* = 0.030**	r = 0.00011 *p* = 1.0	r = 0.50 *p* = 0.12	r = 0.52 *p* = 0.10
2	T0	CN、JP、KR、SE AT、FR、AU、US PE	r = 0.63 *p* = 0.25	**r = 0.92 *p* = 0.0041**	r = −0.62 *p* = 0.59	r = −0.39 *p* = 0.48
3	T1	HZ、CN、JP、KR DK、SE、AT、FR AU、US、PE	r = −0.49 *p* = 0.25	r = −0.21 *p* = 0.60	r = 0.29 *p* = 0.62	r = 0.15 *p* = 0.75
4	T2	HZ、JP、KR、DK SE、AT、FR、AU US、PE	r = −0.33 *p* = 0.40	r = −0.37 *p* = 0.46	r = 0.26 *p* = 0.62	r = −0.49 *p* = 0.41
5	T3	CN、JP、AT、FR	r = 0.95 *p* = 0.25	r = 0.84 *p* = 0.46	r = −0.59 *p* = 0.62	r = 0.21 *p* = 0.79
6	T4	CN、JP、KR、DK SE、AT、FR、AU US、PE	r = −0.54 *p* = 0.25	r = −0.34 *p* = 0.46	r = 0.31 *p* = 0.62	r = −0.49 *p* = 0.41
7	T5	HZ、CN、JP、KR DK、SE、AT、FR AU、US、PE	r = −0.38 *p* = 0.34	r = 0.093 *p* = 0.79	r = 0.18 *p* = 0.68	r = 0.36 *p* = 0.48
8	T6	CN、JP、KR、DK SE、AT、FR、AU US	r = 0.46 *p* = 0.33	r = 0.42 *p* = 0.46	r = −0.35 *p* = 0.62	r = 0.29 *p* = 0.59
9	T7	HZ、CN、JP、KR DK、SE、AT、FR AU、US、PE	r = 0.086 *p* = 0.80	r = 0.39 *p* = 0.46	r = −0.035 *p* = 0.92	r = −0.54 *p* = 0.41

Figure S19 Relationship between average RA of BSHs with WHO released phenotypes in 11 populations. Correlation that shown significant difference between **a** average RA of BSH and death rate (per 100,000 individuals) of diabetes; **b** average RA of BSH-T0 and death rate (per 100,000 individuals) of cardiovascular diseases (CVD); **c** Correlation between different phylotypes of BSH abundance and death rate of diabetes and CVD, mean blood cholesterol and Body mass index (BMI) of obesity. R and p values were calculated form Pearson correlations to assess the relationship.

Here, the information of the WHO released phenotypes could be found in Table S10. RA, relative abundance.

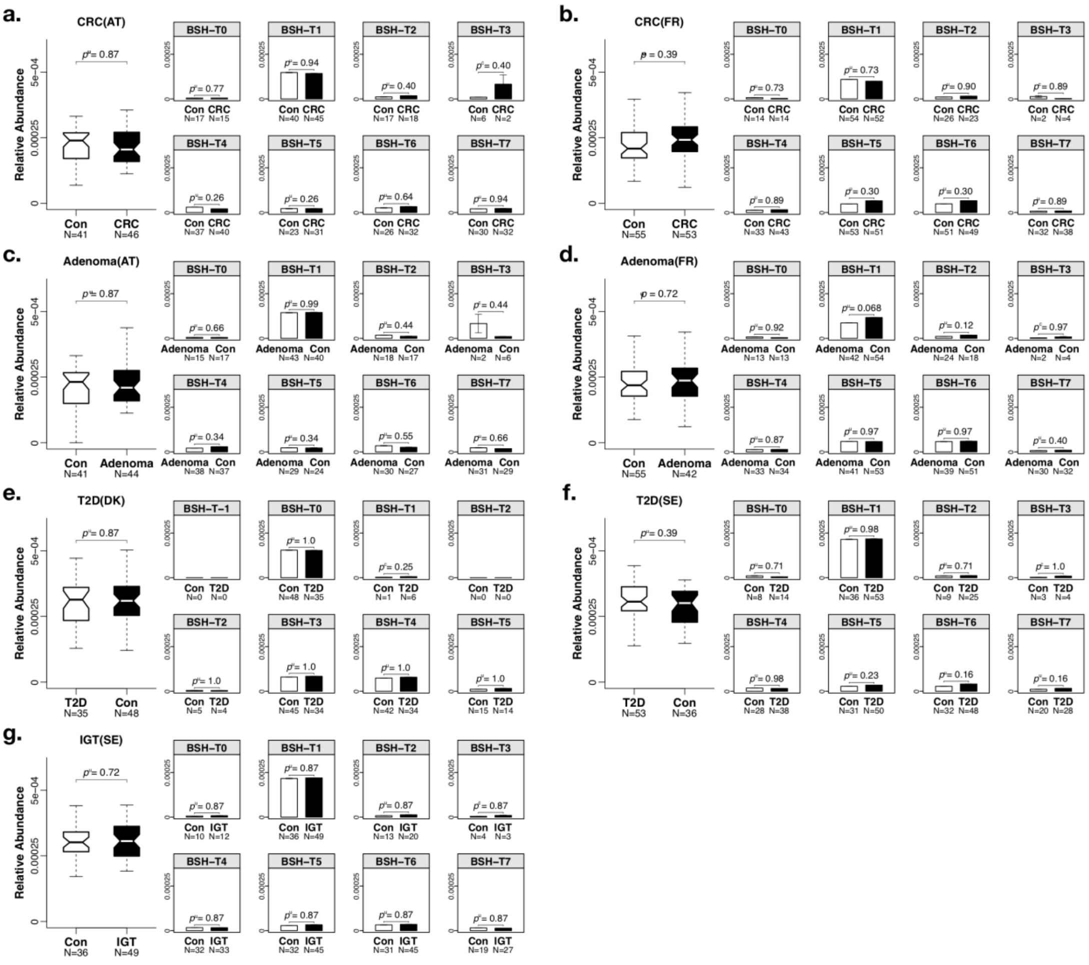

Figure S20 The relative abundance of BSHs among case-control cohorts. a colorectal cancer (CRC) in Austria; **b** colorectal cancer (CRC) in Denmark; **c** Adenoma in Austria; **d** Adenoma in Denmark; **e** Type 2 diabetes (T2D) in Denmark; **f** Type 2 diabetes (T2D) in Sweden; **g** impaired glucose tolerance (IGT) in Sweden. p^c values were calculated by the Chi-squared test; p^u values were calculated by the Mann-Whitney U test, p values in each type were analyzed by followed by false discovery rate correction. The populations from CN and CN^2 are independent cases. Details are shown in Table S3.

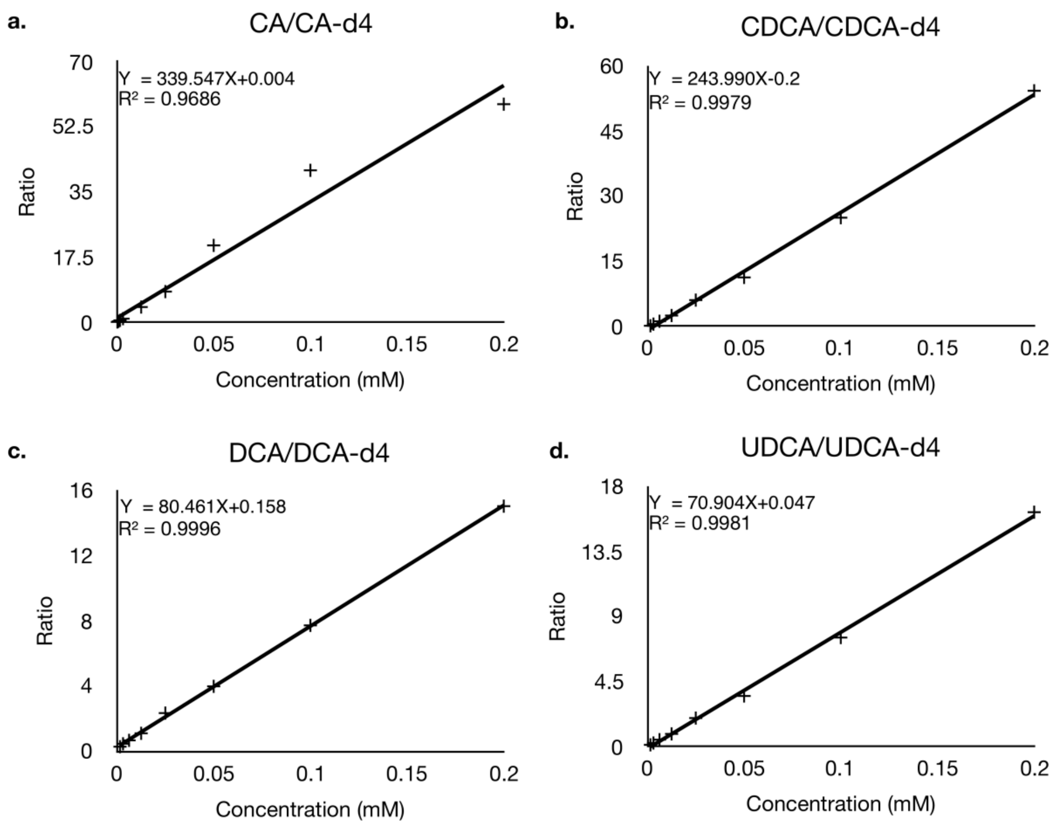

Figure S21 Standard curve of bile acids used in LC-MS/MS. a CA; **b** CDCA; **c** DCA; **d** UDCA.

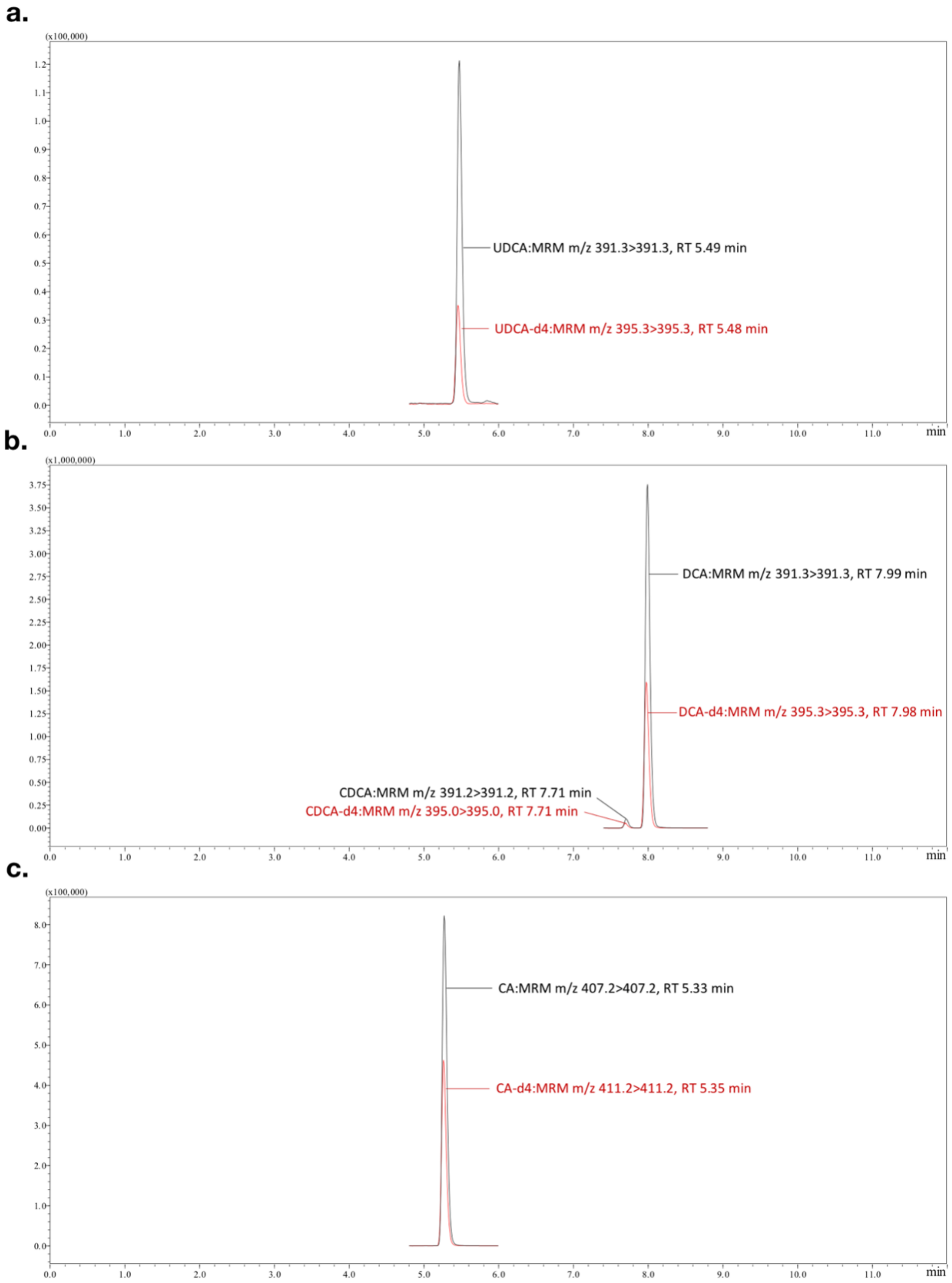

Figure S22 Typical LC spectrum of different bile acids. a UDCA; **b** DCA and CDCA; **c** CA.
